# Adverse childhood experiences and their differential relationships with transdiagnostic mental health outcomes in young adults

**DOI:** 10.1017/S0033291725000893

**Published:** 2025-05-22

**Authors:** Yufan Chen, Zoe Aitken, Dylan Hammond, Andrew Thompson, Steven Marwaha, Chris Davey, Michael Berk, Patrick McGorry, Andrew Chanen, Barnaby Nelson, Aswin Ratheesh

**Affiliations:** 1 Centre for Youth Mental Health, University of Melbourne, Parkville, VIC, Australia; 2 Centre for Health Policy, Melbourne School of Population and Global Health, University of Melbourne, Parkville, VIC, Australia; 3 Orygen, Parkville, VIC, Australia; 4 Division of Mental Health and Wellbeing, Warwick Medical School, University of Warwick; 5 Institute for Mental Health, University of Birmingham, Birmingham, UK; 6 Department of Psychiatry, University of Melbourne, Parkville, VIC, Australia; 7 Institute for Mental and Physical Health and Clinical Translation (IMPACT), Deakin University, Barwon Health, Geelong, VIC, Australia; 8 Discipline of Psychiatry and Mental Health, University of New South Wales, Sydney, NSW, Australia

**Keywords:** abuse, adverse childhood experiences, ALSPAC, anxiety, depression, effect modification, mental disorders, mental health, neglect, psychiatry, psychosis

## Abstract

Adverse childhood experiences (ACEs) are associated with poor mental health outcomes, which are increasingly conceptualized from a transdiagnostic perspective. We examined the impact of ACEs on transdiagnostic mental health outcomes in young adulthood and explored potential effect modification. We included participants from the Avon Longitudinal Study of Parents and Children with prospectively measured data on ACEs from infancy till age 16 as well as mental health outcomes at ages 18 and 24. Exposures included emotional neglect, bullying, and physical, sexual or emotional abuse. The outcome was a pooled transdiagnostic Stage of 1b (subthreshold but clinically significant symptoms) or greater level (Stage 1b+) of depression, anxiety, or psychosis – a clinical stage typically associated with first need for mental health care. We conducted multivariable logistic regressions, with multiple imputation for missing data. We explored effect modification by sex at birth, first-degree family history of mental disorder, childhood neurocognition, and adolescent personality traits. Stage 1b + outcome was associated with any ACE (OR = 2.66, 95% CI = 1.68–4.22), any abuse (OR = 2.08, 95% CI = 1.38–3.14), bullying (OR = 2.15, 95% CI = 1.43–3.24), and emotional neglect (OR = 1.68, 95% CI = 1.06–2.67). Emotional neglect had a weaker association with the outcome among females (OR = 1.14, 95% CI = 0.61–2.14) than males (OR = 3.49, 95% CI = 1.64–7.42) and among those with higher extraversion (OR = 0.91, 95% CI = 0.85–0.97), in unweighted (*n* = 2,126) and weighted analyses (*n* = 7,815), with an openness–neglect interaction observed in the unweighted sample. Sex at birth, openness, and extraversion could modify the effects of adverse experiences, particularly emotional neglect, on the development of poorer transdiagnostic mental health outcomes.

## Introduction

Adverse childhood experiences (ACEs) include maltreatment (e.g. abuse), interpersonal loss (e.g. parental separation), family dysfunction (e.g. inter-parental conflict), and other childhood adversities (e.g. socio-economic hardship) occurring in childhood and adolescence (Sahle et al., 2022). These experiences are common, with reported estimates between 20% and 70% among adults worldwide (Daníelsdóttir et al., 2024; Grummitt, Baldwin, Lafoa’i, Keyes, & Barrett, 2024; Madigan et al., 2025; McKay et al., 2021; Sahle et al., 2022). ACEs have been classified into broad categories, such as maltreatment, interpersonal loss, family dysfunction, and others, in a recent umbrella review (Sahle et al., 2022). Childhood maltreatment, including physical and sexual abuse, emotional abuse, and neglect, has been most frequently associated with anxiety, depression, and suicidality (Sahle et al., 2022).

ACEs are non-specific risk factors that confer a 2- to 6-fold increase in the odds of developing any mental disorder in adulthood (Abate et al., 2024; Sahle et al., 2022). Experiencing multiple ACEs (Merrick, Ford, Ports, & Guinn, 2018) is associated with higher odds of later mental health problems (Hughes et al., 2017), with some indications of a ‘dose-effect’ (Daníelsdóttir et al., 2024). Explanations for such associations include ACEs leading to heightened attention toward threats, and maladaptive schemas triggering negative cognitions, anxious behavior, emotional dysregulation, depressed mood, and paranoia (Thompson et al., 2011). This indicates that ACEs confer a predisposition to poor outcomes transdiagnostically. Several major mental health conditions appear to have common risk factors and shared treatments in earlier stages (Dalgleish, Black, Johnston, & Bevan, 2020; Shah et al., 2020), suggesting that transdiagnostic prevention approaches might have advantages over single-disorder approaches. Furthermore, while previous studies have investigated the associations between ACEs and discrete mental health outcomes, such as depression, anxiety, and psychosis (Sahle et al., 2022), understanding the relationship between ACEs and a pooled transdiagnostic outcome might help overcome the challenge of achieving statistical power to reliably detect low-incidence outcomes (Cuijpers, 2003). This approach might also offer public health advantages, as potential interventions for transdiagnostic risk factors might have benefits across many disorders simultaneously.

While existing prospective studies have consistently shown that ACEs increase the odds of mental disorders in adulthood, several studies retrospectively ascertained exposure to ACEs from official court and child protection records (McKay et al., 2021; Sahle et al., 2022). Such methods only included the most severe and/or reported instances and likely underestimated the role of more concealed experiences, such as emotional neglect (McKay et al., 2021), which is less likely to receive attention from welfare agencies (Chamberland, Fallon, Black, Nico, & Chabot, 2012). Prospectively collected, self-report data are needed to study the potential impacts of all ACEs.

In such data, investigating factors that moderate the associations between ACEs and mental disorders could identify vulnerable subgroups. Markers of additional vulnerability when being exposed to ACEs have had limited study, in contrast to the literature on protective factors. While prior, predominantly cross-sectional studies have indicated the mediating (Buchanan, Walker, Boden, Mansoor, & Newton-Howes, 2023; Zhao et al., 2022) or moderating (Crouch, Radcliff, Strompolis, & Srivastav, 2018) effect of protective factors, such as psychological processes, family support, and ethnicity (Elkins, Kassenboehmer, & Schurer, 2017), there is little direct evidence for interaction effects from prospective studies that can guide the identification of youth at higher risk. Clinical risk factors typically assessed by general practitioners and primary care clinicians, such as sex or gender, family history of mental disorders, childhood symptoms, and adolescent personality traits, may be particularly relevant. Examining whether these factors increase the risk of poor mental health outcomes when exposed to ACEs could be used to select participants in preventive intervention trials, and subsequently, for selective prevention. For instance, parental mental disorders might influence children’s mental health trajectories via early exposure to dysfunctional family environments, whereas supportive family environments might mitigate the effects of losses (Adjei et al., 2024; Behere, Basnet, & Campbell, 2017; Kamis, 2021; Sahle et al., 2022). Females may be more vulnerable to interpersonal abuse and are more likely to develop mental health symptoms (Garcia et al., 2016; Herringa et al., 2013; Sternberg et al., 1993). Personality traits measured within the five-factor model, or related markers, such as self-esteem and self-regulation, have also been hypothesized or identified to interact with ACEs to contribute to poorer mental health outcomes, predominantly in cross-sectional studies (Gallardo-Pujol & Pereda, 2013; Kushner, Bagby, & Harkness, 2017; Rogosch & Cicchetti, 2004; Vinkers et al., 2014). The development of such personality traits may also be influenced by ACEs and in turn affect the way youth engage with or adapt to ACEs (Mallet, 2022). Finally, lower neurocognitive functioning is associated with early exposure to adversity (Melby et al., 2020), and higher neurocognitive functioning might protect against developing psychopathology (Fares-Otero et al., 2024). To our knowledge, no study, to date, has examined interactions between these commonly clinically assessed effect modifiers, ACEs, and transdiagnostic mental health outcomes, using prospective data. Prospective studies examining interactions are vital as mental health outcomes including mood states could affect the report of effect modifiers such as personality traits (Hibbert, 2018).

This study, using the Avon Longitudinal Study of Parents and Children (ALSPAC; Boyd et al., 2013; Fraser et al., 2013), had two aims. First, to investigate the prospective associations between adverse childhood experiences and transdiagnostic mental health outcomes in young adulthood that are likely to need clinical mental health care. We focused on depressive, anxiety, and psychotic symptoms, operationalized as a pooled transdiagnostic outcome (Ratheesh et al., 2023). Second, we examined the role of family history of mental disorder in a first-degree relative, sex at birth, childhood neurocognition, and personality traits in adolescence as potential effect modifiers of the association between ACEs and mental health outcomes in young adulthood.

## Method

We used a prospective cohort design, adhering to the Strengthening the Reporting of Observational Studies in Epidemiology (STROBE) statement (Supplementary Table 1). Informed consent for the use of data collected via questionnaires and clinics was obtained from participants following the recommendations of the ALSPAC Ethics and Law Committee at the time.

### Data source

This study used data from the ALSPAC cohort. Pregnant women resident in Avon, UK with expected dates of delivery between 1st April 1991 and 31st December were invited to take part (Boyd et al., 2013; Fraser et al., 2013). The initial number of pregnancies enrolled was 14,541, and 13,988 children were alive at 1 year of age. Children who were unable to join the study at age 1 were invited to participate in the study during adolescence and adulthood bringing the cumulative total to 15,546 participants. A proportion of children, their mothers, and families have been followed since the prenatal period, with recurring measurements at different timepoints throughout the children’s lives (Fraser et al., 2013; Major-Smith et al., 2023; Northstone et al., 2019). Through self-reported questionnaires, interviews, and clinical data, ALSPAC investigators measured parents and children’s personal experiences, household, and neighborhood environment, health outcomes and other factors throughout childhood, adolescence, and young adulthood. Approximately 14–56% of the total eligible participants with data on the exposures and outcomes at ages 16, 18, and 24 years formed the analytic sample (Supplementary Figure 1).

The study website contains details of all the data available through a fully searchable data dictionary and variable search tool and reference the following webpage: http://www.bristol.ac.uk/alspac/researchers/our-data/. Please see Supplementary Methods for detailed description of the recruitment.

### Exposure

ACEs were assessed via self-report questionnaires. Children began self-reporting their experiences from age 8, before which their mother/mother’s partner completed the questionnaires, recalling events over a 12-month period. Data from these questionnaires were integrated into exposures from birth to age 16 into 19 different ACEs (Supplementary Figure 2; Houtepen, Heron, Suderman, Tilling, & Howe, 2018). We focused on the five ACEs linked to maltreatment including physical abuse (defined as ‘adult in family was ever physically cruel toward or hurt the child’), sexual abuse (‘the child was ever sexually abused, forced to perform sexual acts or touch someone in a sexual way’), emotional abuse (‘parent was ever emotionally cruel toward the child or often said hurtful/insulting things to the child’), emotional neglect (‘child always felt excluded, misunderstood or never important to family, parents never asked or never listened when child talked about their free time’), and bullying (‘child was a victim of bullying on a weekly basis’; Houtepen et al., 2018). Rather than broader environmental factors, we chose maltreatment-related ACEs as they primarily stem from interpersonal interactions and have the strongest relationship with mental health outcomes (Fitzgerald & Bishop, 2024). While all 19 ACEs could be examined, we limited our examination to maltreatment ACEs as testing many interactions could identify spurious associations from Type I error (Ranganathan, Pramesh, & Buyse, 2016). Dichotomous responses across multiple timepoints from birth to age 16 were used to derive binary constructs as developed by Houtepen et al. (2018). We created pooled variables that described ‘any ACE’, ‘cumulative ACE’ (classified as having experienced one, two, or three or more ACEs), and ‘any abuse’, which described physical, sexual, or emotional abuse, consistent with the categorization in the CDC-Kaiser Permanente ACE Study (Felitti et al., 1998), with details in the Supplementary Methods.

### Outcome

Given the strong inter-relationships and shared causal factors among anxiety, depressive, and psychotic symptoms reported in our previous work (Ratheesh et al., 2023), we considered a pooled transdiagnostic stage-based outcome including these symptom types during the transitional years of ages 18 and 24. In transdiagnostic staging approaches, Stage 1a represents nonspecific or mild symptoms, Stage 1b suggests subthreshold but clinically significant symptoms, and Stages 2, 3, and 4 suggest full threshold discrete disorders at varying levels of severity and persistence (Shah et al., 2020). Our primary outcome was Stage 1b or higher (1b+), representing at least moderate symptoms associated with functional impact. By this stage, young people show specific clinically relevant mental disorder symptoms that signal the need for mental health intervention (Ratheesh et al., 2023).

Participant-reported anxiety, depressive, and psychotic symptoms were used to generate the pooled outcome. Briefly, depression and anxiety were assessed using the Clinical Interview Schedule-Revised (CIS-R), with Stage 1b + defined as at least a) recurrent or persistent moderate symptoms at ages 18 and 24, or b) severe symptoms with functional impairment at either age 18 or 24 (Ratheesh et al., 2023). Psychosis was examined by the Psychosis Like Symptoms Interviews, with Stage 1b + characterized by at least monthly primary psychotic symptoms associated with distress, functional impairment, or help-seeking within the past 6 months at either age 18 or 24. The pooled Stage 1b + outcome was considered present if at least one Stage 1b + outcome was present and considered absent, if symptoms were absent for all three disorders. Please see the Supplementary Methods and our previous report for detailed definition and rationale for this outcome (Ratheesh et al., 2023).

While broader transdiagnostic stage outcomes have included manic symptoms, substance use, and externalizing symptoms (Hickie et al., 2013), it may be unwise to pool these in a simple dichotomous outcome (Ratheesh et al., 2023), as we have previously argued. Such an outcome could have substantial heterogeneity, which could obscure associations or interaction effects.

### Covariates

#### Potential confounders

We selected confounders a priori, using a Directed Acyclic Graph (Supplementary Figure 3) outlining hypothesized causal pathways. We included four confounders measured during pregnancy (i.e. prior to ACEs), consistent with previous studies investigating the associations between ACEs and mental health outcomes (McKay et al., 2021). Confounders included child’s ethnicity, sex at birth, maternal age at delivery, and household social class (indicated by mother’s and partner’s occupational background).

#### Effect modifiers

We examined the role of sex at birth, first-degree family history of severe mood and psychotic disorders, childhood neurocognition, and adolescent personality traits. These variables have been found to be related to early adversity and mental health outcome, as previously discussed. The child’s sex was determined at birth, dichotomized into female and male. First-degree family history of mental disorders was based on report of parents having experienced severe depression or schizophrenia as reported by parents from birth until children were 8 years of age (or until parents were approximately 35 years of age), ensuring that at least a majority of the risk for depression and psychosis was captured. We included the full-scale intelligence quotient (FSIQ) scores measured by the Wechsler Intelligence Scale for Children at age 8. Personality traits were measured at age 14 using computer-assisted self-assessment using the International Personality Item Pool (Goldberg, 1999), assessing for the ‘Big Five’ personality factors in continuous scores: openness (or intellect vs unconventionality), conscientiousness, extraversion, agreeableness, and emotional stability (or neuroticism). These are further described in the Supplementary Methods.

### Statistical analysis

To account for missing data, we conducted multiple imputation (MI) using multivariate imputation by chained equations. MI models included all analysis variables and auxiliary variables (described in the Supplementary Methods). In our analytic sample, there were approximately 0–20% missing observations for the variables in the primary analyses (Supplementary Table 2), therefore, we generated 20 datasets with data imputed using the ‘mi estimate’ command.

We described sample characteristics using a randomly selected imputed dataset. Univariable and multivariable logistic regression models were fitted to examine the association between each ACE and young adults’ Stage 1b + mental health outcome, adjusting for confounders.

To test for effect modification, we included interaction terms between any abuse, emotional neglect, and bullying and each effect modifier in separate statistical models. We used *F*-test statistics to test for interaction and obtained stratum-specific odds ratios (ORs) for categorical effect modifiers (i.e. sex and first-degree family history of severe mood and psychotic disorders) and ORs associated with each unit increase in numerical effect modifiers (i.e. childhood neurocognition and adolescent personality traits). The Benjamini–Hochberg procedure was applied to correct for multiple testing (Lee & Lee, 2018). Additive and multiplicative interactions were examined to quantify the deviation of joint effect from the sum and the product of the individual effects of each exposure and effect modifier (VanderWeele & Knol, 2014). We used the ‘nlcom’ command to calculate the relative excess risk due to interaction (RERI) to address additive interaction and the ratio of odds ratios (ROR) derived from the multivariable regression coefficients to quantify multiplicative interaction (VanderWeele & Knol, 2014). We described the strength of interaction using VanderWeele’s interaction continuum for interactions between exposures and each categorical effect modifier (VanderWeele, 2019). This allows identified interactions to be placed on a rank indicating their strength. A higher rank (numerically lower) indicates a higher strength of association. The model and the ranks are detailed in the Supplementary Methods.

*Sensitivity and post-hoc analyses:* For ease of interpretation, we described effect modification where personality scores were categorized into tertiles (low, normal, high; Supplementary Table 3) and FSIQ scores were categorized into low (the bottom 25%), normal (the middle 50%), and high (the top 25%; Supplementary Table 3), in line with the previous research (Ayers, Gulley, & Verghese, 2020; Moran, Klinteberg, Batty, & Vågerö, 2009).

We replicated all main analyses using a combined approach of inverse-probability weighting and multiple imputation (IPW/MI; Seaman, White, Copas, & Li, 2012), with auxiliary variables and confounders measured at birth or in gestation with less than 10% missingness to generate weights for larger missing data blocks, to examine whether the findings were replicated in a larger proportion of the eligible participants.

We replicated our analyses using the complete case sample to check for similarity with imputed findings, and we examined associations between each ACE and each mental disorder outcome (Stage 1b + of depression, anxiety, and psychosis) to identify outliers. We also conducted post-hoc analyses to examine whether excluding each symptom type from the pooled outcome altered the results of the statistically significant (*p* < .05) multivariable logistic regressions and effect modification tests. Finally, given the possibility that the data could depart from missing at random (MAR) assumptions (Supplementary Table 2), we conducted post-hoc sensitivity analysis for statistically significant effect modification results using the method developed by Carpenter, Kenward, and White (2007)). The overall parameter estimate was computed as a weighted average of the MAR-based estimates, with the weight determined by the coefficient for the association between having the outcome and the likelihood of having complete data for any ACE.

All statistical analyses were conducted using Stata BE/18.

## Results

Among 15,645 eligible pregnancies, 2,126 participants had complete data for both the exposure (i.e. any abuse, emotional neglect, and bullying) and the pooled Stage 1b + mental health outcome (Supplementary Figure 1). The complete case sample was impacted by selective attrition with differences in sex, ethnicity, social class, and family history of mental health conditions. In post-hoc analyses using IPW/MI, the weighted sample (*N* = 7,815) was similar to the initial eligible sample in most characteristics except for lower social class (Supplementary Table 2). In the complete case sample, most participants were female (60.07%), white (96.28%), from high social class (60.11%), and 56.02% participants had experienced at least one ACE (Table 1). 10–20% of observations of data were missing in our primary analyses (Table 1 & Supplementary Table 2), and all ACEs were independent of covariates (Supplementary Table 4). The prevalence of Stage 1b + outcome was 5.32% (*n* = 113).

**Table 1.** Prevalence of each variable as well as multivariable logistic regressions for the association between each exposure or covariate and the Stage 1b + mental health outcome^a^

	Overall sample (*n* = 2,126) No.(%)	Stage 1b + outcome in young adulthood (*n* = 113) No.(%)	Not meeting Stage 1b + outcome in young adulthood (*n* = 2,013) No.(%)				
				Unadjusted OR (95% CI)	*p*-Value	Adjusted OR^b^ (95% CI)	*p*-Value
*Exposures*							
Any ACE	1191 (56.02)	87 (76.99)	1104 (54.84)	2.56 (1.62–4.05)	<.001	2.66 (1.68–4.22)	<.001
Cumulative ACE							
1	736 (34.62)	38 (33.63)	698 (34.67)	1.69 (0.97–2.92)	.062	1.76 (1.01–3.04)	.045
2	338 (15.90)	35 (30.97)	303 (15.05)	3.86 (2.24–6.63)	<.001	4.05 (2.34–7.01)	<.001
3+	117 (5.50)	14 (12.39)	103 (5.12)	4.50 (2.23–9.10)	<.001	4.64 (2.28–9.45)	<.001
Any abuse	697 (32.78)	55 (48.67)	642 (31.89)	2.10 (1.40–3.15)	<.001	2.08 (1.38–3.14)	<.001
Physical abuse	402 (18.91)	41 (36.28)	361 (17.93)	2.48 (1.58–3.88)	<.001	2.53 (1.60–3.98)	<.001
Sexual abuse	94 (4.42)	9 (7.96)	85 (4.22)	2.16 (1.06–4.42)	.034	1.89 (0.92–3.88)	.085
Emotional abuse	410 (19.29)	37 (32.74)	373 (18.53)	2.02 (1.29–3.16)	.002	2.00 (1.27–3.14)	.003
Emotional neglect	370 (17.40)	26 (23.01)	344 (17.09)	1.63 (1.03–2.57)	.038	1.68 (1.06–2.67)	.027
Bullying	508 (23.89)	43 (38.05)	465 (23.10)	2.00 (1.34–3.00)	.001	2.15 (1.43–3.24)	<.001
*Covariates*							
Social class							
Low social class (manual and non-manual skilled, partly skilled or unskilled)	848 (39.89)	50 (44.25)	798 (39.64)	1.17 (0.79–1.75)	.429	1.14 (0.75–1.74)	.525
High social class (professional or managerial and technical)	1278 (60.11)	63 (55.75)	1215 (60.36)	Reference	Reference	Reference	Reference
Ethnicity							
Non-white (Black Caribbean, Black African, other black, Indian, Pakistani, Bangladeshi, Chinese and other)	79 (3.72)	7 (6.19)	72 (3.58)	1.55 (0.65–3.69)	.320	1.58 (0.66–3.77)	.300
White	2047 (96.28)	106 (93.81)	1941 (96.42)	Reference	Reference	Reference	Reference
First-degree family history of mood and psychotic disorder							
Present	388 (18.25)	34 (30.09)	354 (17.59)	2.16 (1.39–3.36)	.001	2.09 (1.33–3.28)	.001
Absent	1738 (81.75)	79 (69.91)	1659 (82.41)	Reference	Reference	Reference	Reference
Sex							
Female	1277 (60.07)	83 (73.45)	1194 (59.31)	1.90 (1.24–2.91)	.003	1.89 (1.23–2.90)	.004
Male	849 (42.11)	30 (26.55)	819 (40.69)	Reference	Reference	Reference	Reference
*Covariates*							
Maternal age (years)	29.77 (4.43)	29.65 (4.39)	29.78 (4.44)	1.00 (0.95–1.04)	.865	1.00 (0.96–1.05)	.890
Personality (openness)	36.39 (5.42)	38.27 (6.56)	36.29 (5.33)	1.06 (1.02–1.10)	.005	1.07 (1.02–1.11)	.002
Personality (conscientiousness)	32.36 (5.83)	30.17 (5.82)	32.49 (5.81)	0.93 (0.90–0.96)	<.001	0.93 (0.90–0.97)	<.001
Personality (extraversion)	35.34 (7.06)	35.00 (8.22)	35.36 (6.99)	0.99 (0.96–1.02)	.633	0.99 (0.96–1.02)	.387
Personality (agreeableness)	38.57 (5.09)	40.21 (5.62)	38.48 (5.04)	1.06 (1.02–1.11)	.004	1.05 (1.00–1.10)	.030
Personality (emotional stability)	32.02 (6.56)	26.81 (7.60)	32.32 (6.37)	0.88 (0.85–0.91)	<.001	0.88 (0.85–0.92)	<.001
Neurocognition	109.06 (15.56)	105.55 (16.06)	109.25 (15.51)	0.99 (0.98–1.00)	.106	0.99 (0.98–1.00)	.183

a Using a randomly selected MI dataset (*n* = 2,126).

b Confounders include maternal age, social class, ethnic group, and sex.

Having experienced any ACE led to a 2.66-fold increase in the odds of the Stage 1b + outcome (95% CI = 1.68–4.22, *p* < .001, Table 1). Among different ACEs, physical abuse had the strongest association with the outcome (OR = 2.53, 95% CI = 1.60–3.98, *p* < .001), followed by bullying (OR = 2.08, 95% CI = 1.43–3.24, *p* < .001), emotional abuse (OR = 2.00, 95% CI = 1.27–3.14, *p* = .003), and emotional neglect (OR = 1.68, 95% CI = 1.06–2.67, *p* = .027). There was no evidence of an association between the Stage 1b + outcome and sexual abuse (OR = 1.89, 95% CI = 0.92–3.88). A ‘dose-effect’ was evident, such that people who had experienced three or more ACEs (OR = 4.46, 95% CI = 2.28–9.45, *p* < .001) or two ACEs (OR = 4.05, 95% CI = 2.34–7.01, *p* < .001) had higher odds of the Stage 1b + outcome compared to those experiencing one type of ACE (OR = 1.76, 95% CI = 1.01–3.04, *p* = .045). Associations between different ACEs and Stage 1b + of individual disorders indicated similar patterns (Supplementary Table 5).

### Effect modification

#### First-degree family history of mental disorders and childhood neurocognition

There was no evidence of effect modification by first-degree family history of mental disorders or childhood neurocognition on the association between the Stage 1b + mental health outcome and any abuse, emotional neglect, or bullying (*p*-values for *F*-tests for interaction >.05; Table 2).

**Table 2.** Logistic regression models between the exposures and the outcome across levels of each effect modifier^a^

Outcome	Exposure	OR (95% CI)	*F*-test	*p*-Value	BH corrected threshold
Stage 1b+	Any abuse				
	Sex^b^			.708	.208
	Female	2.18 (1.34–3.57)			
	Male	1.83 (0.86–3.93)			
	Family history of mental disorder			.371	.135
	Present	1.46 (0.69–3.06)			
	Absence	2.19 (1.34–3.58)			
	Openness		1.01 (0.93–1.09)	.876	.240
	Conscientiousness		1.01 (0.94–1.08)	.810	.219
	Extraversion		0.97 (0.91–1.03)	.375	.146
	Agreeableness		0.95 (0.87–1.05)	.301	.115
	Emotional stability		0.96 (0.89–1.03)	.291	.104
	Neurocognition		0.99 (0.96–1.02)	.442	.156
	Emotional neglect				
	Sex^b^			**.027**	**.031^c^**
	Female	1.14 (0.61–2.14)			
	Male	3.49 (1.64–7.42)			
	Family history of mental disorder			.870	.229
	Present	1.56 (0.65–3.78)			
	Absence	1.71 (0.97–3.01)			
	Openness		0.84 (0.77–0.92)	**.0002**	**.010^c^**
	Conscientiousness		1.00 (0.92–1.09)	.962	.250
	Extraversion		0.91 (0.85–0.97)	**.004**	**.021^c^**
	Agreeableness		0.90 (0.81–1.00)	.051	.040
	Emotional stability		1.04 (0.97–1.12)	.259	.083
	Neurocognition		0.98 (0.95–1.01)	.204	.063
	Bullying				
	Sex^b^			.686	.188
	Female	2.27 (1.40–3.68)			
	Male	1.89 (0.88–4.04)			
	Family history of mental disorder (present)			.259	.083
	Present	2.98 (1.42–6.25)			
	Absence	1.76 (1.06–2.94)			
	Openness		0.93 (0.86–1.01)	.105	.052
	Conscientiousness		0.98 (0.91–1.07)	.702	.198
	Extraversion		1.01 (0.95–1.08)	.670	.177
	Agreeableness		0.98 (0.89–1.07)	.637	.167
	Emotional stability		0.96 (0.89–1.04)	.311	.125
	Neurocognition		0.98 (0.96–1.01)	.248	.073

a Confounders include maternal age, social class, ethnic group, and sex except in note b below.

b Confounders include maternal age, social class, and ethnic group.

c *p*-value of the *F*-test is smaller than the critical Benjamini–Hochberg (BH) corrected threshold.

The bolded text indicates that they are below the conventional statistical significance threshold of 0.05. They are in bold to aid readability and highlight significant findings.

#### Sex at birth

There was evidence for sex modifying the effect of emotional neglect on the Stage 1b + outcomes (*p* = .027, threshold = 0.031, Table 2). The association was weak among females (OR = 1.14, 95% CI = 0.61–2.14) but was stronger and statistically significant among males (OR = 3.49, 95% CI = 1.64–7.42, *p* = .001; Table 3). The negative multiplicative (ROR = 0.33, 95% CI = 0.12–0.88, *p* < .05) and non-significant additive interaction between emotional neglect and female sex (Table 3) was ranked fourth in VanderWeele’s interaction continuum (2019) for two causative exposures. This indicates a weak interaction.

**Table 3.** Logistic regression models between the exposures and the outcome across levels of the dichotomous effect modifiers including sex and first-degree family history of mental disorders

	First-degree family history of severe depression and psychotic disorder^a^					
	Presence				Absence	
	OR (95% CI)	*p*-Value	ROR	RERI (95% CI)	OR (95% CI)	*p*-Value
Any abuse	1.46 (0.69–3.06)	.319	0.67 (0.27–1.62)	−0.12 (−2.44–2.20)	2.19 (1.34–3.58)	.002
Emotional neglect	1.56 (0.65–3.78)	.321	0.91 (0.31–2.68)	0.51 (−2.40–3.43)	1.71 (0.97–3.01)	.063
Bullying	2.98 (1.42–6.25)	.004	1.69 (0.68–4.22)	2.50 (−0.44–5.43)	1.76 (1.06–2.94)	.030

	**Sex^b^**					
	Female				Male	
	OR (95% CI)	*p*-Value	ROR	RERI (95% CI)	OR (95% CI)	*p*-Value
Any abuse	2.18 (1.34–3.57)	.002	1.19 (0.48–2.96)	1.23 (−0.56–3.02)	1.83 (0.86–3.93)	.118
Emotional neglect	1.14 (0.61–2.14)	.674	**0.33 (0.12–0.88)^c^**	−2.10 (−5.28–1.08)	3.49 (1.64–7.42)	.001
Bullying	2.27 (1.40–3.68)	.001	1.20 (0.49–2.97)	1.50 (−0.58–3.58)	1.89 (0.88–4.04)	.103

*Note:* ROR, ratio of odds ratios; RERI, relative excess risk due to interaction.

a Confounders include maternal age, social class, ethnic group, and sex are adjusted for.

b Confounders include maternal age, social class and ethnic group are adjusted for.

c *p*-value<.05.

The bolded text indicates that they are below the conventional statistical significance threshold of 0.05. They are in bold to aid readability and highlight significant findings.

**Table 4.** Logistic regression models between the exposures and the outcome across levels of the trichotomous effect modifiers, including personality traits and neurocognition^a^

	Openness									
	Low openness (1st tertile)				Normal openness (2nd tertile)		High openness (3rd tertile)			
	OR (95% CI)	*p*-Value	ROR	RERI (95% CI)	OR (95% CI)	*p*-Value	OR (95% CI)	*p*-Value	ROR	RERI (95% CI)
Any abuse	2.18 (0.90–5.30)	.085	1.26 (0.39–4.05)	0.08 (−1.58–1.74)	1.73 (0.83–3.61)	.145	2.35 (1.29–4.29)	.005	1.36 (0.53–3.51)	1.84 (−0.61–4.29)
Emotional neglect	4.89 (2.05–11.68)	<.001	3.02 (0.77–11.84)	**2.13 (0.27–4.00)**^**b**^	1.62 (0.63–4.16)	.313	0.66 (0.23–1.92)	.443	0.41 (0.09–1.79)	−2.82 (−5.77–0.12)
Bullying	2.92 (1.27–6.75)	.012	1.61 (0.51–5.11)	0.61 (−1.19–2.40)	1.82 (0.80–3.98)	.134	2.04 (1.06–3.92)	.033	1.12 (0.40–3.12)	1.11 (−1.80–4.02)

	Conscientiousness									
	Low conscientiousness (1st tertile)				Normal conscientiousness (2nd tertile)		High conscientiousness (3rd tertile)			
	OR (95% CI)	*p*-Value	ROR	RERI (95% CI)	OR (95% CI)	*p*-Value	OR (95% CI)	*p*-Value	ROR	RERI (95% CI)
Any abuse	2.38 (1.28–4.40)	.006	1.85 (0.67–5.15)	1.18 (−0.58–2.94)	1.28 (0.59–2.79)	.528	3.23 (1.12–9.36)	.031	2.52 (0.62–10.26)	0.28 (−1.09–1.65)
Emotional neglect	1.55 (0.79–3.07)	.204	0.86 (0.29–2.51)	0.23 (−1.71–2.19)	1.82 (0.81–4.07)	.148	1.38 (0.38–4.95)	.625	0.76 (0.16–3.50)	−0.38 (−1.85–1.08)
Bullying	2.08 (1.13–3.83)	.018	1.06 (0.39–2.92)	0.76 (−1.08–2.60)	1.96 (0.92–4.20)	.083	1.66 (0.52–5.24)	.389	0.85 (0.20–3.52)	−0.55 (−2.12–1.03)

	Extraversion									
	Low extraversion (1st tertile)				Normal extraversion (2nd tertile)		High extraversion (3rd tertile)			
	OR (95% CI)	*p*-Value	ROR	RERI (95% CI)	OR (95% CI)	*p*-Value	OR (95% CI)	*p*-Value	ROR	RERI (95% CI)
Any abuse	2.85 (1.35–6.02)	.006	1.76 (0.58–5.34)	1.08 (−0.71–2.86)	1.61 (0.76–3.42)	.213	1.95 (0.94–4.04)	.072	1.21 (0.43–3.41)	−0.09 (−1.69–1.51)
Emotional neglect	2.63 (1.29–5.37)	.008	2.21 (0.79–6.19)	1.44 (−0.42–3.29)	1.19 (0.60–2.38)	.621	NA	NA	NA	NA
Bullying	1.66 (0.79–3.48)	.181	0.77 (0.26–2.27)	−0.54 (−2.47–1.40)	2.14 (1.01–4.53)	.047	2.83 (1.32–6.08)	.008	1.32 (0.44–4.02)	0.79 (−1.01–2.60)

The bolded text indicates that they are below the conventional statistical significance threshold of 0.05. They are in bold to aid readability and highlight significant findings.

**Table 5.** Logistic regression models between the exposures and the outcome across levels of the trichotomous effect modifiers, including personality traits and neurocognition (continued)^a^

	Agreeableness									
	Low agreeableness (1st tertile)				Normal agreeableness (2nd tertile)		High agreeableness (3rd tertile)			
	OR (95% CI)	*p*-Value	ROR	RERI (95% CI)	OR (95% CI)	*p*-Value	OR (95% CI)	*p*-Value	ROR	RERI (95% CI)
Any abuse	2.43 (0.89–6.65)	.085	1.21 (0.34–4.33)	0.29 (−1.51–2.09)	2.01 (0.98–4.12)	.057	2.04 (1.08–3.88)	.029	1.02 (0.38–2.73)	0.83 (−1.45–3.11)
Emotional neglect	4.89 (2.01–11.91)	<.001	4.63 (1.27–16.85)^b^	2.21 (0.26–4.16)^b^	1.06 (0.40–2.76)	.911	1.12 (0.43–2.91)	.819	1.06 (0.25–4.40)	−0.94 (−3.70–1.81)
Bullying	2.98 (1.26–7.05)	.013	1.48 (0.49–4.46)	0.85 (−1.25–2.93)	2.02 (0.89–4.14)	.056	2.03 (1.04–3.95)	.038	1.00 (0.37–2.75)	0.73 (−2.00–3.48)

	Emotional stability									
	Low emotional stability (1st tertile)				Normal emotional stability (2nd tertile)		High emotional stability (3rd tertile)			
	OR (95% CI)	*p*-Value	ROR	RERI (95% CI)	OR (95% CI)	*p*-Value	OR (95% CI)	*p*-Value	ROR	RERI (95% CI)
Any abuse	2.08 (1.19–3.64)	.010	1.18 (0.43–3.22)	2.78 (−0.51–6.07)	1.77 (0.77–4.07)	.178	1.59 (0.58–4.38)	.366	0.90 (0.24–3.34)	−0.40 (−2.04–1.24)
Emotional neglect	1.53 (0.83–2.81)	.175	1.27 (0.35–4.58)	1.36 (−1.90–4.62)	1.20 (0.40–3.58)	.746	1.97 (0.63–6.13)	.241	1.65 (0.34–8.03)	0.37 (−1.47–2.22)
Bullying	2.10 (1.23–3.60)	.007	2.90 (0.81–10.36)	**2.93 (0.15–5.71)^b^**	0.72 (0.23–2.26)	.578	1.85 (0.57–6.00)	.306	2.55 (0.51–12.88)	0.11 (−1.82–2.04)

	Neurocognition									
	Low neurocognition (1st tertile)				Normal neurocognition (2nd tertile)		High neurocognition (3rd tertile)			
	OR (95% CI)	*p*-Value	ROR	RERI (95% CI)	OR (95% CI)	*p*-Value	OR (95% CI)	*p*-Value	ROR	RERI (95% CI)
Any abuse	2.35 (1.10–5.00)	.027	1.11 (0.41–3.04)	0.64 (−1.18–2.45)	2.11 (1.12–3.97)	.021	1.69 (0.75–3.82)	.208	0.80 (0.29–2.23)	−0.41 (−2.04–1.21)
Emotional neglect	2.82 (1.32–6.02)	.008	1.76 (0.59–5.26)	1.66 (−0.32–3.64)	1.60 (0.75–3.42)	.224	0.51 (0.12–2.25)	.376	0.32 (0.06–1.75)	−1.71 (−3.34–0.07)^b^
Bullying	2.83 (1.39–5.77)	.004	1.11 (0.43–2.85)	1.15 (−0.79–3.09)	2.55 (1.37–4.73)	.003	0.98 (0.37–2.61)	.972	0.39 (0.12–1.24)	−1.68 (−3.51–0.16)

*Note: RERI, relative excess risk due to interaction; ROR, ratio of odds ratios.*

a Confounders include maternal age, social class, ethnic group, and sex.

b *p*-value <.05.

NA, not applicable as model did not converge due to low cell numbers.

#### Personality measured during adolescence

There was strong evidence for effect modification by openness on the association between emotional neglect and the Stage 1b + outcome (*p* < .001, Benjamini–Hochberg corrected threshold = 0.010, Table 2). For each score increase in openness, there was a 0.84-fold decrease in the odds of experiencing the outcome associated with emotional neglect (95% CI = 0.77–0.92, *p* < 0.05, Table 2). Post-hoc analysis indicated that compared to those with normal openness (OR = 1.62, 95% CI = 0.63–4.16, *p* = .313), the association between the outcome and emotional neglect was stronger and statistically significant among those with low openness (OR = 4.89, 95% CI = 2.05–11.68, *p* < .001; Table 4). The effect of low openness was ranked second on the interaction continuum for two causative exposures (VanderWeele, 2019), suggesting a strong interaction.

There was also evidence for effect modification by extraversion on the association between emotional neglect and the Stage 1b + outcome (95% CI = 0.85–0.97, *p* = .004, Benjamini–Hochberg corrected threshold = .021, Table 2). For each score increase in extraversion, there was a 0.91-fold decrease in the odds of experiencing the Stage 1b + outcome. Post-hoc analysis indicated that those with low extraversion had a higher risk of the Stage 1b + outcome associated with emotional neglect (OR = 2.63, 95% CI 1.29–5.37, *p* = 0.08) while those with normal extraversion did not (OR = 1.19, 95% CI = 0.60–2.38, *p* = .621; Table 3). The model did not converge for high extraversion.

### Sensitivity and post-hoc analysis

Using IPW/MI to extend our findings to roughly half the eligible sample (*N* = 7,815), the interaction findings for sex and extraversion remained significant, but the impact of low openness on outcomes was attenuated. In sensitivity and post-hoc analyses using a delta-adjusted method for departures from MAR assumptions, findings for the association between ACEs and the Stage 1b + outcome (Supplementary Table 6) and for effect modification by sex, openness and extraversion on the impact of emotional neglect (Supplementary Tables 7–8b) were similar to that with the analytic sample. Results using complete case data were similar to the findings using MI data (Supplementary Tables 5–8). Post-hoc sensitivity analyses excluding each mental health symptom type from the transdiagnostic outcome indicated a similar pattern of interactions (Supplementary Tables 9–10b).

## Discussion

In this prospective study, we confirmed that ACEs were associated with an increased risk of poor transdiagnostic mental health outcomes in young adulthood. We also identified that youth who experienced emotional neglect prior to age 16 were more likely to experience poorer mental health outcomes in young adulthood if they were male or had lower self-reported openness or extraversion in adolescence. Lower extraversion and male sex remained relevant in roughly half the total eligible sample in weighted analyses.

Our results contribute to a growing body of literature identifying ACEs as both a common and major contributor to mental ill-health. In our sample, approximately 60% of participants had experienced at least one ACE, consistent with previous studies (McKay et al., 2021). Experiencing ACEs increased the odds of negative mental health outcomes, with evidence of greater association with multiple types of ACEs. While broadly consistent with existing findings, the magnitude of the association (ORs between 1 and 3) in our results was slightly lower than some existing studies (ORs between 2 and 6; Daníelsdóttir et al., 2024; Grummitt et al., 2024; McKay et al., 2021; Sahle et al., 2022). This might be explained by different appraisals of ACEs observed between retrospective and prospective studies (Baldwin, Coleman, Francis, & Danese, 2024).

We found that sex modifies the effect of emotional neglect, with males showing increased vulnerability to mental ill health in young adulthood. The use of different coping techniques might explain the higher resilience shown by females in response to emotional neglect (Brown, Fite, Stone, Richey, & Bortolato, 2018; McGloin & Widom, 2001). Females tend to use emotion-focused strategies and to seek social support, whereas males tend to employ problem-focused strategies (Brown et al., 2018). While both problem- and emotion-focused coping strategies reduced mental disorder symptoms, the latter led to a faster decrease, and such improvements were particularly pronounced among females (Fluharty, Bu, Steptoe, & Fancourt, 2021). Future studies could consider coping styles and other mechanisms such as self-regulation (Rollins & Crandall, 2021) or self-esteem (Kim, Lee, & Park, 2022) in conferring resilience to specific ACEs, considering between-sex differences.

There was also evidence that higher extraversion, and in a sub-sample higher openness, in adolescence might be protective against the impact of emotional neglect. This reflects the known association between resilience and these traits (Campbell-Sills, Cohan, & Stein, 2006; Nakaya, Oshio, & Kaneko, 2006; Oshio, Taku, Hirano, & Saeed, 2018). Resilience depends on positive interpretation of adversity (Hähnchen, 2022). When facing negative emotions and maladaptive thoughts induced by adversity, extraverted individuals cultivate resilience through cognitive restructuring, as they tend to focus on solving the problem itself rather than the associated negative affect (Connor-Smith & Flachsbart, 2007). Extraversion facets, such as expressiveness and positive emotionality (Burgin et al., 2012; Riggio & Riggio, 2002), also support resilience, mitigating the harm associated with ACEs (Hähnchen, 2022). Overall, we identified evidence for interaction between several effect modifiers with emotional neglect but none with other types of abuse. This could be explained by abuse being a more potent risk factor indicated by the magnitude of association from our data and from prior literature (Connor-Smith & Flachsbart, 2007; Gardner, Thomas, & Erskine, 2019; Li, D’Arcy, & Meng, 2016; Liu et al., 2017; Taillieu, Brownridge, Sareen, & Afifi, 2016). It suggests that emotional neglect might have specific pathways to mental health problems.

The threat-deprivation model (McLaughlin Sheridan, & Lambert, 2014) provides some explanation. Emotional neglect and passive maltreatment, which constitutes deprivation, differs from experiences that involve actual (i.e. physical and sexual abuse, and bullying) or implied harm (i.e. emotional abuse) leading to the perception of threat. Threat exposure is believed to directly and strongly influence emotion regulation and reaction to potential danger through fear learning and conditioning, whereas deprivation could preferentially affect cognition through lack of experiences and pruning of under-stimulated synaptic connections (McLaughlin et al., 2014; Sheridan, Peverill, Finn, & McLaughlin, 2017). Prior studies have indicated that deprivation-related ACEs had a weaker impact on internalizing symptoms (e.g. anxiety and depression; Henry et al., 2021; Uddin et al., 2024) but higher risk of executive functioning difficulties (Johnson et al., 2021). When facing a relatively weak effect, higher openness and extraversion could lead to youth seeking novel experiences and social integration (Yu, Zhao, Li, Zhang, & Li, 2021), while those with lower openness and extraversion might be unable to do so, potentially worsening the impact of such ACEs over time. Conversely, early-life threat exposure could cause harm irrespective of personality traits, as threats could cause strong impacts on social–emotional processing and difficulty disengaging from negative experiences (McLaughlin & Lambert, 2017). This signals the need to develop different interventions targeting children exposed to deprivation (Sheridan & McLaughlin, 2016).

It should also be noted that exposure to ACEs may have a reciprocal relationship with the development of openness and extraversion (Fosse & Holen, 2007; Grusnick, Garacci, Eiler, Williams, & Egede, 2020; Pos et al., 2016), yet such traits could prompt individuals’ exposure to additional ACEs (Mallett, 2022) through their engagement with new environments (Shiner & Caspi, 2003). Therefore, caution should be taken when interpreting the interplay between ACEs and personality, which may contribute to the observed associations. While openness and extraversion are malleable in childhood and early adolescence (Branje, van Lieshout, & Gerris, 2007; Tetzner, Becker, & Bihler, 2023), they are associated with minimal change from mid adolescence to young adulthood (ages 15–24; Elkins et al., 2017; Vecchione, Alessandri, Barbaranelli, & Caprara, 2012). This suggests that such traits could be used to select youth at higher risk of additional harm in the face of emotional neglect.

We did not find evidence for effect modification by first-degree family history of mental disorder. While these children might be predisposed to adverse mental health outcomes due to genetic risks and the impact of impaired parenting (Burke, 2003; Mattejat & Remschmidt, 2008; van Santvoort Hosman, van Doesum & Janssens, 2014), some develop protective compensatory mechanisms and resilience through coping (Pölkki Ervast, & Huupponen 2004), or by caring for their parent with a mental illness (McDougall, O’Connor, & Howell, 2018; van der Mijl & Vingerhoets, 2017). Variable responses to maltreatment (Mattejat & Remschmidt, 2008) could have led to the lack of significant interactions.

We did not find that neurocognition moderated the impact of ACEs. This could be because the differences between participants regarding their cognitive processes may be explained better by their engagement with intellectual experiences (openness) rather than intellectual functioning (Ziegler, Cengia, Mussel, & Gerstorf, 2015). While openness encourages the pursuit of intellectual experience, crystalized intelligence fosters positive experience of successful problem-solving, subsequently enhancing motivation and skills for further growth (Ziegler et al., 2015).

This study’s primary limitation is the smaller proportion of the eligible sample due to longitudinal attrition, which is common among birth cohort studies. This selective attrition might reduce the generalizability of our findings to non-white individuals and those with parents from non-professional or managerial backgrounds. However, additional analyses in a weighted sample including half the total eligible participants identified similar findings with respect to sex and extraversion, suggesting acceptable generalizability. The finding regarding openness needs confirmation in prospective studies with less attrition. Second, we could not examine the possibility that early life ACEs could influence the personality traits studied in this investigation due to our use of time-collapsed ACE variables. However, this allowed us to comprehensively examine the role of ACEs, which are likely to have a cumulative impact on adult mental health outcomes. Third, we acknowledge that children might engage in help-seeking behavior such as undergoing counselling in response to ACEs, which would subsequently affect the outcome. However, we did not include this in our analysis as ALSPAC only captured help-seeking for specific difficulties such as self-harm. There were also challenges in temporality with young people potentially receiving counselling at several points in adolescence or young adulthood. Additionally, we utilized sex at birth, which limited our capacity to investigate the role of self-determined gender on the risks associated with ACEs. This was, however, necessary to inform the confounding effects of sex on early-life exposures. Finally, evidence of statistical interaction must be interpreted with caution, given the high possibility of detecting a range of interactions within the sufficient component causal model of complex mental disorders (Zammit, Lewis, Dalman, & Allebeck, 2010). Hence, we corrected for multiple comparisons and interpreted findings on the interaction continuum, highlighting the modifying effect of sex and extraversion on the association between emotional neglect and the Stage 1b + outcome as the most promising finding. Our findings are also likely robust as we used prospectively collected data from birth to adulthood. We ensured temporality between ACEs and young adults’ mental health outcomes, supporting conclusions regarding their causative role in the development of mental disorder. ALSPAC data collection utilized widely used and validated clinical questionnaires to identify outcomes, confounders, and effect modifiers. The use of a transdiagnostic approach allowed us to identify previously unrecognized impacts of ACEs on subgroups, such as males and those with certain personality characteristics. Finally, our results were robust to sensitivity analysis excluding individual symptom types from our transdiagnostic outcome.

## Conclusions

Our findings suggest the need for future research to identify why some children might have differential risk, aiming to identify malleable risk factors. Our results, as well as prior data, suggest that all children exposed to, or at-risk of, maltreatment should be offered preventive interventions such as parent training. In addition, there is a need to develop public health interventions addressing emotional neglect, potentially targeting subgroups or malleable mechanisms.

## Supporting information

Chen et al. supplementary material 1 Chen et al. supplementary material

Chen et al. supplementary material 2 Chen et al. supplementary material

Chen et al. supplementary material 3 Chen et al. supplementary material

## Supplementary material

The supplementary material for this article can be found at http://doi.org/10.1017/S0033291725000893.

## References

[r1] Abate, B. B., Sendekie, A. K., Merchaw, A., Abebe, G. K., Azmeraw, M., Alamaw, A. W., Zemariam, A. B., Kitaw, T. A., Kassaw, A., Wodaynew, T., Kassie, A. M., Yilak, G., & Kassa, M. A. (2024). Adverse childhood experiences are associated with mental health problems later in life: An umbrella review of systematic review and meta-analysis. Neuropsychobiology, 84(1), 48–64. 10.1159/000542392 39557030

[r2] Adjei, N. K., Schlüter, D. K., Melis, G., Straatmann, V. S., Fleming, K. M., Wickham, S., Munford, L., McGovern, R., Howard, L. M., Kaner, E., Wolfe, I., & Taylor-Robinson, D. C. (2024). Impact of parental mental health and poverty on the health of the next generation: A multi-trajectory analysis using the UK millennium cohort study. The Journal of Adolescent Health: Official Publication of the Society for Adolescent Medicine, 74(1), 60–70. 10.1016/j.jadohealth.2023.07.029 37831048

[r3] Ayers, E., Gulley, E., & Verghese, J. (2020). The effect of personality traits on risk of incident pre-dementia syndromes. Journal of the American Geriatrics Society, 68(7), 1554–1559. 10.1111/jgs.16424 32488931 PMC7363540

[r4] Baldwin, J. R., Coleman, O., Francis, E. R., & Danese, A. (2024). Prospective and Retrospective Measures of Child Maltreatment and Their Association With Psychopathology: A systematic review and meta-analysis. JAMA Psychiatry, 81(8), 769–781. 10.1001/jamapsychiatry.2024.0818 38691376 PMC11063927

[r5] Behere, A. P., Basnet, P., & Campbell, P. (2017). Effects of family structure on mental health of children: A preliminary study. Indian Journal of Psychological Medicine, 39(4), 457–463. 10.4103/0253-7176.211767 28852240 PMC5559994

[r6] Boyd, A., Golding, J., Macleod, J., Lawlor, D. A., Fraser, A., Henderson, J., Molloy, L., Ness, A., Ring, S., & Davey Smith, G. (2013). Cohort Profile: The ‘children of the 90s’--the index offspring of the Avon Longitudinal Study of Parents and Children. International Journal of Epidemiology, 42(1), 111–127. 10.1093/ije/dys064 22507743 PMC3600618

[r7] Branje, S. J. T., van Lieshout, C. F. M., & Gerris, J. R. M. (2007). Big five personality development in adolescence and Adulthood. European Journal of Personality, 21(1), 45–62. 10.1002/per.596

[r8] Brown, S., Fite, P. J., Stone, K., Richey, A., & Bortolato, M. (2018). Associations between emotional abuse and neglect and dimensions of alexithymia: The moderating role of sex. Psychological Trauma: Theory, Research, Practice and Policy, 10(3), 300–308. 10.1037/tra0000279 28414491 PMC5645215

[r9] Buchanan, M., Walker, G., Boden, J. M., Mansoor, Z., & Newton-Howes, G. (2023). Protective factors for psychosocial outcomes following cumulative childhood adversity: Systematic review. BJPsych Open, 9(6), e197. 10.1192/bjo.2023.561 37855106 PMC10594245

[r10] Burgin, C. J., Brown, L. H., Royal, A., Silvia, P. J., Barrantes-Vidal, N., & Kwapil, T. R. (2012). Being with others and feeling happy: Emotional expressivity in everyday life. Personality and Individual Differences, 53(3), 185–190. 10.1016/j.paid.2012.03.006 27013770 PMC4803035

[r11] Burke, L. (2003). The impact of maternal depression on familial relationships. International Review of Psychiatry (Abingdon, England), 15(3), 243–255. 10.1080/0954026031000136866 15276963

[r12] Campbell-Sills, L., Cohan, S. L., & Stein, M. B. (2006). Relationship of resilience to personality, coping, and psychiatric symptoms in young adults. Behaviour Research and Therapy, 44(4), 585–599. 10.1016/j.brat.2005.05.001 15998508

[r13] Carpenter, J. R., Kenward, M. G., & White, I. R. (2007). Sensitivity analysis after multiple imputation under missing at random: A weighting approach. Statistical Methods in Medical Research, 16(3), 259–275. 10.1177/0962280206075303 17621471

[r14] Chamberland, C., Fallon, B., Black, T., Nico, T., & Chabot, M. (2012). Correlates of substantiated emotional maltreatment in the second Canadian incidence study. Journal of Family Violence, 27(3), 201–213. 10.1007/s10896-012-9414-8

[r15] Connor-Smith, J. K., & Flachsbart, C. (2007). Relations between personality and coping: A meta-analysis. Journal of Personality and Social Psychology, 93(6), 1080–1107. 10.1037/0022-3514.93.6.1080 18072856

[r16] Crouch, E., Radcliff, E., Strompolis, M., & Srivastav, A. (2018). Safe, stable, and nurtured: Protective factors against poor physical and mental health outcomes following exposure to Adverse Childhood Experiences (ACEs). Journal of Child & Adolescent Trauma, 12(2), 165–173. 10.1007/s40653-018-0217-9 32318189 PMC7163854

[r17] Cuijpers, P. (2003). Examining the effects of prevention programs on the incidence of new cases of mental disorders: The lack of statistical power. The American Journal of Psychiatry, 160(8), 1385–1391. 10.1176/appi.ajp.160.8.1385 12900296

[r18] Dalgleish, T., Black, M., Johnston, D., & Bevan, A. (2020). Transdiagnostic approaches to mental health problems: Current status and future directions. Journal of Consulting and Clinical Psychology, 88(3), 179–195. 10.1037/ccp0000482 32068421 PMC7027356

[r19] Daníelsdóttir, H. B., Aspelund, T., Shen, Q., Halldorsdottir, T., Jakobsdóttir, J., Song, H., Lu, D., Kuja-Halkola, R., Larsson, H., Fall, K., Magnusson, P. K. E., Fang, F., Bergstedt, J., & Valdimarsdóttir, U. A. (2024). Adverse childhood experiences and adult mental health outcomes. JAMA Psychiatry, 81(6), 586–594. 10.1001/jamapsychiatry.2024.0039 38446452 PMC10918580

[r20] Elkins, R. K., Kassenboehmer, S. C., & Schurer, S. (2017). The stability of personality traits in adolescence and young adulthood. Journal of Economic Psychology, 60, 37–52. 10.1016/j.joep.2016.12.005

[r21] Fares-Otero, N. E., Borràs, R., Solé, B., Torrent, C., Garriga, M., Serra-Navarro, M., Forte, M. F., Montejo, L., Salgado-Pineda, P., Montoro, I., Sánchez-Gistau, V., Pomarol-Clotet, E., Ramos-Quiroga, J. A., Tortorella, A., Menculini, G., Grande, I., Garcia-Rizo, C., Martinez-Aran, A., Bernardo, M., Pacchiarotti, I., … Verdolini, N. (2024). Cognitive reserve moderates the relationship between childhood maltreatment, objective and subjective cognition, and psychosocial functioning in individuals with first-episode psychosis. Psychological Trauma: Theory, Research, Practice and Policy, 17(3), 520–536. 10.1037/tra0001650 38512168

[r22] Felitti, V. J., Anda, R. F., Nordenberg, D., Williamson, D. F., Spitz, A. M., Edwards, V., Koss, M. P., & Marks, J. S. (1998). Relationship of childhood abuse and household dysfunction to many of the leading causes of death in adults. The Adverse Childhood Experiences (ACE) Study. American Journal of Preventive Medicine, 14(4), 245–258. 9635069 10.1016/s0749-3797(98)00017-8

[r23] Fitzgerald, M., & Bishop, A. (2024). Challenging the use of the overall adverse childhood experiences (ACEs) score: Comparing total ACEs, maltreatment, and household dysfunction on mental health problems among White, African American, and Native American women under correctional control. The American Journal of Orthopsychiatry. Advance online publication. 10.1037/ort0000784 39432352

[r24] Fluharty, M., Bu, F., Steptoe, A., & Fancourt, D. (2021). Coping strategies and mental health trajectories during the first 21 weeks of COVID-19 lockdown in the United Kingdom. Social Science & Medicine (1982), 279, 113958. 10.1016/j.socscimed.2021.113958 33965772 PMC9756769

[r25] Fosse, G. K., & Holen, A. (2007). Reported maltreatment in childhood in relation to the personality features of Norwegian adult psychiatric outpatients. The Journal of Nervous and Mental Disease, 195(1), 79–82. 10.1097/01.nmd.0000252312.98109.d4 17220744

[r26] Fraser, A., Macdonald-Wallis, C., Tilling, K., Boyd, A., Golding, J., Davey Smith, G., Henderson, J., Macleod, J., Molloy, L., Ness, A., Ring, S., Nelson, S. M., & Lawlor, D. A. (2013). Cohort profile: The Avon Longitudinal Study of Parents and Children: ALSPAC mothers cohort. International Journal of Epidemiology, 42(1), 97–110. 10.1093/ije/dys066 22507742 PMC3600619

[r27] Gallardo-Pujol, D., & Pereda, N. (2013). Person-environment transactions: Personality traits moderate and mediate the effects of child sexual victimization on psychopathology. Personality and Mental Health, 7(2), 102–113. 10.1002/pmh.1192 24343936

[r28] Garcia, M., Montalvo, I., Creus, M., Cabezas, Á., Solé, M., Algora, M. J., Moreno, I., Gutiérrez-Zotes, A., & Labad, J. (2016). Sex differences in the effect of childhood trauma on the clinical expression of early psychosis. Comprehensive Psychiatry, 68, 86–96. 10.1016/j.comppsych.2016.04.004 27234188

[r29] Gardner, M. J., Thomas, H. J., & Erskine, H. E. (2019). The association between five forms of child maltreatment and depressive and anxiety disorders: A systematic review and meta-analysis. Child Abuse & Neglect, 96, 104082. 10.1016/j.chiabu.2019.104082 31374447

[r30] Goldberg, L. R. (1999). A broad-bandwidth, public domain personality inventory measuring the lower-level facets of several five-factor models. In I. Mervielde, I. Deary, F. De Fruyt, & F. Ostendorf (Eds.), Personality psychology in Europe, 7, 7–28. Tilburg, The Netherlands: Tilburg University Press.

[r31] Grummitt, L., Baldwin, J. R., Lafoa’i, J., Keyes, K. M., & Barrett, E. L. (2024). Burden of mental disorders and suicide attributable to childhood maltreatment. JAMA Psychiatry, 81(8), 782–788. 10.1001/jamapsychiatry.2024.0804 38717764 PMC11079790

[r32] Grusnick, J. M., Garacci, E., Eiler, C., Williams, J. S., & Egede, L. E. (2020). The association between adverse childhood experiences and personality, emotions and affect: Does number and type of experiences matter?. Journal of Research in Personality, 85, 103908. 10.1016/j.jrp.2019.103908 32863469 PMC7453784

[r33] Hähnchen, D. (2022). The relationship between extraversion and resilience during the Covid-19 pandemic among university students: Does cognitive restructuring partially mediate its association? (Bachelor’s thesis, University of Twente). University of Twente Student Theses. https://essay.utwente.nl/91368/

[r34] Henry, L. M., Gracey, K., Shaffer, A., Ebert, J., Kuhn, T., Watson, K. H., Gruhn, M., Vreeland, A., Siciliano, R., Dickey, L., Lawson, V., Broll, C., Cole, D. A., & Compas, B. E. (2021). Comparison of three models of adverse childhood experiences: Associations with child and adolescent internalizing and externalizing symptoms. Journal of Abnormal Psychology, 130(1), 9–25. 10.1037/abn0000644 33271039 PMC8687696

[r35] Herringa, R. J., Birn, R. M., Ruttle, P. L., Burghy, C. A., Stodola, D. E., Davidson, R. J., & Essex, M. J. (2013). Childhood maltreatment is associated with altered fear circuitry and increased internalizing symptoms by late adolescence. Proceedings of the National Academy of Sciences of the United States of America, 110(47), 19119–19124. 10.1073/pnas.1310766110 24191026 PMC3839755

[r36] Hibbert, A. (2018). The influence of mood on self-reported personality (T). University of British Columbia. Retrieved from https://open.library.ubc.ca/collections/ubctheses/24/items/1.0375851

[r37] Hickie, I. B., Scott, E. M., Hermens, D. F., Naismith, S. L., Guastella, A. J., Kaur, M., Sidis, A., Whitwell, B., Glozier, N., Davenport, T., Pantelis, C., Wood, S. J., & McGorry, P. D. (2013). Applying clinical staging to young people who present for mental health care. Early Intervention in Psychiatry, 7(1), 31–43. 10.1111/j.1751-7893.2012.00366.x 22672533

[r38] Houtepen, L. C., Heron, J., Suderman, M. J., Tilling, K., & Howe, L. D. (2018). Adverse childhood experiences in the children of the Avon Longitudinal Study of Parents and Children (ALSPAC). Wellcome Open Research, 3, 106. 10.12688/wellcomeopenres.14716.1 30569020 PMC6281007

[r91] Hughes, K., Bellis, M. A., Hardcastle, K. A., Sethi, D., Butchart, A., Mikton, C., Jones, L., & Dunne, M. P. (2017). The effect of multiple adverse childhood experiences on health: a systematic review and meta-analysis. The Lancet. Public health, 2(8), e356–e366. 10.1016/S2468-2667(17)30118-4 29253477

[r39] Johnson, D., Policelli, J., Li, M., Dharamsi, A., Hu, Q., Sheridan, M. A., McLaughlin, K. A., & Wade, M. (2021). Associations of early-life threat and deprivation with executive functioning in childhood and adolescence: A systematic review and meta-analysis. JAMA Pediatrics, 175(11), e212511. 10.1001/jamapediatrics.2021.2511 34309651 PMC8314173

[r40] Kamis, C. (2021). The long-term impact of parental mental health on children’s distress trajectories in adulthood. Society and Mental Health, 11(1), 54–68. 10.1177/2156869320912520 34094696 PMC8172076

[r42] Kim, Y., Lee, H., & Park, A. (2022). Patterns of adverse childhood experiences and depressive symptoms: Self-esteem as a mediating mechanism. Social Psychiatry and Psychiatric Epidemiology, 57(2), 331–341. 10.1007/s00127-021-02129-2 34191037 PMC8243305

[r44] Kushner, S. C., Bagby, R. M., & Harkness, K. L. (2017). Stress generation in adolescence: Contributions from five-factor model (FFM) personality traits and childhood maltreatment. Personality Disorders, 8(2), 150–161. 10.1037/per0000194 27213513

[r45] Lee, S., & Lee, D. K. (2018). What is the proper way to apply the multiple comparison test?. Korean Journal of Anesthesiology, 71(5), 353–360. 10.4097/kja.d.18.00242 30157585 PMC6193594

[r46] Li, M., D’Arcy, C., & Meng, X. (2016). Maltreatment in childhood substantially increases the risk of adult depression and anxiety in prospective cohort studies: Systematic review, meta-analysis, and proportional attributable fractions. Psychological Medicine, 46(4), 717–730. 10.1017/S0033291715002743 26708271

[r47] Liu, J., Fang, Y., Gong, J., Cui, X., Meng, T., Xiao, B., He, Y., Shen, Y., & Luo, X. (2017). Associations between suicidal behavior and childhood abuse and neglect: A meta-analysis. Journal of Affective Disorders, 220, 147–155. 10.1016/j.jad.2017.03.060 28623759

[r48] Madigan, S., Thiemann, R., Deneault, A. A., Fearon, R. M. P., Racine, N., Park, J., Lunney, C. A., Dimitropoulos, G., Jenkins, S., Williamson, T., & Neville, R. D. (2025). Prevalence of adverse childhood experiences in child population samples: A systematic review and meta-analysis. JAMA Pediatrics, 179(1), 19–33. 10.1001/jamapediatrics.2024.4385 39527072 PMC11555579

[r49] Major-Smith, D., Heron, J., Fraser, A., Lawlor, D. A., Golding, J., & Northstone, K. (2023). The Avon Longitudinal Study of Parents and Children (ALSPAC): A 2022 update on the enrolled sample of mothers and the associated baseline data. Wellcome Open Research, 7, 283. 10.12688/wellcomeopenres.18564.1 37664415 PMC10472060

[r50] Mallett, C. A. (2022). *Adverse childhood experiences impacting psychological well-being and cardiac autonomic dysregulation: Examining openness to experience as a resilience factor* (Publication No. 29400039) [Doctoral dissertation, Howard University]. ProQuest Dissertations & Theses Global. https://www.proquest.com/dissertations-theses/adverse-childhood-experiences-impacting/docview/2805388910/se-2

[r51] Mattejat, F., & Remschmidt, H. (2008). The children of mentally ill parents. Deutsches Arzteblatt International, 105(23), 413–418. 10.3238/arztebl.2008.0413 19626164 PMC2696847

[r52] McDougall, E., O’Connor, M., & Howell, J. (2018). ‘Something that happens at home and stays at home’: An exploration of the lived experience of young carers in Western Australia. Health & Social Care in the Community, 26(4), 572–580. 10.1111/hsc.12547 29457295

[r53] McGloin, J. M., & Widom, C. S. (2001). Resilience among abused and neglected children grown up. Development and Psychopathology, 13(4), 1021–1038. 10.1017/s095457940100414x 11771905

[r54] McKay, M. T., Cannon, M., Chambers, D., Conroy, R. M., Coughlan, H., Dodd, P., Healy, C., O’Donnell, L., & Clarke, M. C. (2021). Childhood trauma and adult mental disorder: A systematic review and meta-analysis of longitudinal cohort studies. Acta Psychiatrica Scandinavica, 143(3), 189–205. 10.1111/acps.13268 33315268

[r55] McLaughlin, K. A., & Lambert, H. K. (2017). Child trauma exposure and psychopathology: Mechanisms of risk and resilience. Current Opinion in Psychology, 14, 29–34. 10.1016/j.copsyc.2016.10.004 27868085 PMC5111863

[r90] McLaughlin, K. A., Sheridan, M. A., & Lambert, H. K. (2014). Childhood adversity and neural development: deprivation and threat as distinct dimensions of early experience. Neuroscience and biobehavioral reviews, 47, 578–591. 10.1016/j.neubiorev.2014.10.012 25454359 PMC4308474

[r56] Melby, L., Indredavik, M. S., Løhaugen, G., Brubakk, A. M., Skranes, J., & Vik, T. (2020). Is there an association between full IQ score and mental health problems in young adults? A study with a convenience sample. BMC Psychology, 8(1), 7. 10.1186/s40359-020-0372-2 32000845 PMC6993501

[r57] Merrick, M. T., Ford, D. C., Ports, K. A., & Guinn, A. S. (2018). Prevalence of adverse childhood experiences from the 2011-2014 behavioral risk factor surveillance system in 23 States. JAMA Pediatrics, 172(11), 1038–1044. 10.1001/jamapediatrics.2018.2537 30242348 PMC6248156

[r58] Moran, P., Klinteberg, B. A., Batty, G. D., & Vågerö, D. (2009). Childhood intelligence predicts hospitalization with personality disorder in adulthood: Evidence from a population-based study in Sweden. Journal of Personality Disorders, 23(5), 535–540. 10.1521/pedi.2009.23.5.535 19817633

[r59] Nakaya, M., Oshio, A., & Kaneko, H. (2006). Correlations for Adolescent Resilience Scale with big five personality traits. Psychological Reports, 98(3), 927–930. 10.2466/pr0.98.3.927-930 16933700

[r60] Northstone, K., Lewcock, M., Groom, A., Boyd, A., Macleod, J., Timpson, N., & Wells, N. (2019). The Avon Longitudinal Study of Parents and Children (ALSPAC): An update on the enrolled sample of index children in 2019. Wellcome Open Research, 4, 51. 10.12688/wellcomeopenres.15132.1 31020050 PMC6464058

[r61] Oshio, A., Taku, K., Hirano, M., & Saeed, G. (2018). Resilience and Big Five personality traits: A meta-analysis. Personality and Individual Differences, 127, 54–60. 10.1016/j.paid.2018.01.048

[r62] Pölkki, P., Ervast, S. A., & Huupponen, M. (2004). Coping and resilience of children of a mentally ill parent. Social Work in Health Care, 39(1–2), 151–163. 10.1300/j010v39n01_10 15774389

[r63] Pos, K., Boyette, L. L., Meijer, C. J., Koeter, M., Krabbendam, L., de Haan, L., & For Group (2016). The effect of childhood trauma and Five-Factor Model personality traits on exposure to adult life events in patients with psychotic disorders. Cognitive Neuropsychiatry, 21(6), 462–474. 10.1080/13546805.2016.1236014 27678148

[r64] Ranganathan, P., Pramesh, C. S., & Buyse, M. (2016). Common pitfalls in statistical analysis: The perils of multiple testing. Perspectives in Clinical Research, 7(2), 106–107. 10.4103/2229-3485.179436 27141478 PMC4840791

[r65] Ratheesh, A., Hammond, D., Gao, C., Marwaha, S., Thompson, A., Hartmann, J., Davey, C., Zammit, S., Berk, M., McGorry, P., & Nelson, B. (2023). Empirically driven transdiagnostic stages in the development of mood, anxiety and psychotic symptoms in a cohort of youth followed from birth. Translational Psychiatry, 13(1), 103. 10.1038/s41398-023-02396-4 36990979 PMC10052262

[r66] Riggio, H. R., & Riggio, R. E. (2002). Emotional expressiveness, extraversion, and neuroticism: A meta-analysis. Journal of Nonverbal Behavior, 26, 195–218.

[r67] Rogosch, F. A., & Cicchetti, D. (2004). Child maltreatment and emergent personality organization: Perspectives from the five-factor model. Journal of Abnormal Child Psychology, 32(2), 123–145. 10.1023/b:jacp.0000019766.47625.40 15164856

[r68] Rollins, E. M., & Crandall, A. (2021). Self-regulation and shame as mediators between childhood experiences and young adult health. Frontiers in Psychiatry, 12, 649911. 10.3389/fpsyt.2021.649911 33935835 PMC8085257

[r69] Sahle, B. W., Reavley, N. J., Li, W., Morgan, A. J., Yap, M. B. H., Reupert, A., & Jorm, A. F. (2022). The association between adverse childhood experiences and common mental disorders and suicidality: An umbrella review of systematic reviews and meta-analyses. European Child & Adolescent Psychiatry, 31(10), 1489–1499. 10.1007/s00787-021-01745-2‘ 33638709

[r70] Seaman, S. R., White, I. R., Copas, A. J., & Li, L. (2012). Combining multiple imputation and inverse-probability weighting. Biometrics, 68(1), 129–137. 10.1111/j.1541-0420.2011.01666.x 22050039 PMC3412287

[r71] Shah, J. L., Scott, J., McGorry, P. D., Cross, S. P. M., Keshavan, M. S., Nelson, B., Wood, S. J., Marwaha, S., Yung, A. R., Scott, E. M., Öngür, D., Conus, P., Henry, C., Hickie, I. B., & International Working Group on Transdiagnostic Clinical Staging in Youth Mental Health (2020). Transdiagnostic clinical staging in youth mental health: A first international consensus statement. World Psychiatry: Official Journal of the World Psychiatric Association (WPA), 19(2), 233–242. 10.1002/wps.20745 32394576 PMC7215079

[r72] Sheridan, M. A., & McLaughlin, K. A. (2016). Neurobiological models of the impact of adversity on education. Current Opinion in Behavioral Sciences, 10, 108–113. 10.1016/j.cobeha.2016.05.013 29046891 PMC5642918

[r73] Sheridan, M. A., Peverill, M., Finn, A. S., & McLaughlin, K. A. (2017). Dimensions of childhood adversity have distinct associations with neural systems underlying executive functioning. Development and Psychopathology, 29(5), 1777–1794. 10.1017/S0954579417001390 29162183 PMC5733141

[r74] Shiner, R., & Caspi, A. (2003). Personality differences in childhood and adolescence: Measurement, development, and consequences. Journal of Child Psychology and Psychiatry, and Allied Disciplines, 44(1), 2–32. 10.1111/1469-7610.00101 12553411

[r75] Sternberg, K. J., Lamb, M. E., Greenbaum, C., Cicchetti, D., Dawud, S., Cortes, R. M., Krispin, O., & Lorey, F. (1993). Effects of domestic violence on children’s behavior problems and depression. Developmental Psychology, 29(1), 44–52. 10.1037/0012-1649.29.1.44

[r76] Taillieu, T. L., Brownridge, D. A., Sareen, J., & Afifi, T. O. (2016). Childhood emotional maltreatment and mental disorders: Results from a nationally representative adult sample from the United States. Child Abuse & Neglect, 59, 1–12. 10.1016/j.chiabu.2016.07.005 27490515

[r77] Tetzner, J., Becker, M., and Bihler, L.-M. (2023). Personality development in adolescence: Examining big five trait trajectories in differential learning environments. European Journal of Personality 37, 744–764. 10.1177/08902070221121178

[r78] Thompson, A., Sullivan, S., Lewis, G., Zammit, S., Heron, J., Horwood, J., Thomas, K., Gunnell, D., Hollis, C., Wolke, D., & Harrison, G. (2011). Association between locus of control in childhood and psychotic symptoms in early adolescence: Results from a large birth cohort. Cognitive Neuropsychiatry, 16(5), 385–402. 10.1080/13546805.2010.546077 21623488

[r79] Uddin, H., Islam, A., Nahar Lata, L., Nahar, S., Zakir Hossin, M., & Uddin, J. (2024). Associations of threat- and deprivation-related childhood exposures with children’s mental health and flourishing: The moderating role of family resilience. Children and Youth Services Review, 166, 107912. 10.1016/j.childyouth.2024.107912

[r80] van der Mijl, R. C. W., & Vingerhoets, A. (2017). The positive effects of parentification: An exploratory study among students. Psihologijske Teme, 26(2), 417–430. 10.31820/pt.26.2.8

[r81] Van Santvoort, F., Hosman, C., Doesum, K., & Janssens, J. (2014). Children of mentally ill parents participating in preventive support groups: Parental diagnoses and child risk. Journal of Child and Family Studies, 23, 1345–1354. 10.1007/s10826-012-9686-x

[r82] VanderWeele, T., & Knol, M. (2014). A tutorial on interaction. Epidemiology Methods, 3(1), 33–72. 10.1515/em-2013-0005

[r83] VanderWeele, T. J. (2019). The interaction continuum. Epidemiology (Cambridge, Mass.), 30(5), 648–658. 10.1097/EDE.0000000000001054 31205287 PMC6677614

[r84] Vecchione, M., Alessandri, G., Barbaranelli, C., & Caprara, G. (2012). Gender differences in the Big Five personality development: A longitudinal investigation from late adolescence to emerging adulthood. Personality and Individual Differences, 53(6), 740–746. 10.1016/j.paid.2012.05.033

[r85] Vinkers, C. H., Joëls, M., Milaneschi, Y., Kahn, R. S., Penninx, B. W., & Boks, M. P. (2014). Stress exposure across the life span cumulatively increases depression risk and is moderated by neuroticism. Depression and Anxiety, 31(9), 737–745. 10.1002/da.22262 24753162

[r86] Yu, Y., Zhao, Y., Li, D., Zhang, J., & Li, J. (2021). The relationship between Big Five Personality and Social Well-Being of Chinese Residents: The mediating effect of social support. Frontiers in Psychology, 11, 613659. 10.3389/fpsyg.2020.613659 33762985 PMC7982946

[r87] Zammit, S., Lewis, G., Dalman, C., & Allebeck, P. (2010). Examining interactions between risk factors for psychosis. The British Journal of Psychiatry: The Journal of Mental Science, 197(3), 207–211. 10.1192/bjp.bp.109.070904 20807965

[r88] Zhao, Y., Han, L., Teopiz, K. M., McIntyre, R. S., Ma, R., & Cao, B. (2022). The psychological factors mediating/moderating the association between childhood adversity and depression: A systematic review. Neuroscience and Biobehavioral Reviews, 137, 104663. 10.1016/j.neubiorev.2022.104663 35429512

[r89] Ziegler, M., Cengia, A., Mussel, P., & Gerstorf, D. (2015). Openness as a buffer against cognitive decline: The Openness-Fluid-Crystallized-Intelligence (OFCI) model applied to late adulthood. Psychology and Aging, 30(3), 573–588. 10.1037/a0039493 26146885

