# Supplementary material for: Adverse childhood experiences and their differential relationships with transdiagnostic mental health outcomes in young adults
Source: Psychol Med. 2025 May 22;55:e147. doi: 10.1017/S0033291725000893 (PMC12115273; doi:10.1017/S0033291725000893)
Supplement: Chen et al. supplementary material 1 — Chen et al. supplementary material [file S0033291725000893sup001.docx]

**Supplementary Tables**

**Supplementary Table 1.** *STROBE* *Checklist*

|  | Item No | Recommendation | Page  No |
| --- | --- | --- | --- |
| **Title and abstract** | 1 | (*a*) Indicate the study’s design with a commonly used term in the title or the abstract | 1 (Section 1); 1-2 (Section 2) |
|  |  | (*b*) Provide in the abstract an informative and balanced summary of what was done and what was found | 1-2 (Section 2) |
| Introduction | | | |
| Background/rationale | 2 | Explain the scientific background and rationale for the investigation being reported | 3-5 (Section 2) |
| Objectives | 3 | State specific objectives, including any prespecified hypotheses | 5-6 (Section 2) |
| Methods | | | |
| Study design | 4 | Present key elements of study design early in the paper | 6 (Section 2) |
| Setting | 5 | Describe the setting, locations, and relevant dates, including periods of recruitment, exposure, follow-up, and data collection | 6-7 (Section 2) |
| Participants | 6 | (*a*) *Cohort study*—Give the eligibility criteria, and the sources and methods of selection of participants. Describe methods of follow-up  *Case-control study*—Give the eligibility criteria, and the sources and methods of case ascertainment and control selection. Give the rationale for the choice of cases and controls  *Cross-sectional study*—Give the eligibility criteria, and the sources and methods of selection of participants | 5-6 (Section 2) |
|  |  | (*b*) *Cohort study*—For matched studies, give matching criteria and number of exposed and unexposed  *Case-control study*—For matched studies, give matching criteria and the number of controls per case | n/a |
| Variables | 7 | Clearly define all outcomes, exposures, predictors, potential confounders, and effect modifiers. Give diagnostic criteria, if applicable | 7-10 (Section 2) |
| Data sources/ measurement | 8* | For each variable of interest, give sources of data and details of methods of assessment (measurement). Describe comparability of assessment methods if there is more than one group | 7-10 (Section 2) |
| Bias | 9 | Describe any efforts to address potential sources of bias | 10-12 (Section 2) |
| Study size | 10 | Explain how the study size was arrived at | 12 (Section 2) |
| Quantitative variables | 11 | Explain how quantitative variables were handled in the analyses. If applicable, describe which groupings were chosen and why | 10-12 (Section 2) |
|  | | | |
|  | Item No | Recommendation | Page  No |
| Statistical methods | 12 | (*a*) Describe all statistical methods, including those used to control for confounding | 10-11 (Section 2) |
|  |  | (*b*) Describe any methods used to examine subgroups and interactions | 10-11 (Section 2) |
|  |  | (*c*) Explain how missing data were addressed | 11-12 (Section 2) |
|  |  |  |  |
|  |  | (*d*) *Cohort study*—If applicable, explain how loss to follow-up was addressed  *Case-control study*—If applicable, explain how matching of cases and controls was addressed  *Cross-sectional study*—If applicable, describe analytical methods taking account of sampling strategy | n/a |
|  |  |  |  |
|  |  | (*e*) Describe any sensitivity analyses | 11-12 (Section 2) |

Continued on next page

|  | Item No | | Recommendation | Page  No |
| --- | --- | --- | --- | --- |
| Results | | | | |
| Participants | | 13* | (a) Report numbers of individuals at each stage of study—eg numbers potentially eligible, examined for eligibility, confirmed eligible, included in the study, completing follow-up, and analysed | 12 (Section 2) |
|  |  |  | (b) Give reasons for non-participation at each stage | n/a |
|  |  |  | (c) Consider use of a flow diagram | 12 (Section 2) |
| Descriptive data | | 14* | (a) Give characteristics of study participants (eg demographic, clinical, social) and information on exposures and potential confounders | 10(Section 2) |
|  |  |  | (b) Indicate number of participants with missing data for each variable of interest | 10 (Section 2); 12 (Section 2) |
|  |  |  | (c) *Cohort study*—Summarise follow-up time (eg, average and total amount) | 12 (Section 2) |
| Outcome data | | 15* | *Cohort study*—Report numbers of outcome events or summary measures over time | 10 (Section 2) |
|  |  |  | *Case-control study—*Report numbers in each exposure category, or summary measures of exposure | n/a |
|  |  |  | *Cross-sectional study—*Report numbers of outcome events or summary measures | n/a |
| Main results | | 16 | (*a*) Give unadjusted estimates and, if applicable, confounder-adjusted estimates and their precision (eg, 95% confidence interval). Make clear which confounders were adjusted for and why they were included | 12-13 (Section 2) |
|  |  |  | (*b*) Report category boundaries when continuous variables were categorised | 11 (Section 2) |
|  |  |  | (*c*) If relevant, consider translating estimates of relative risk into absolute risk for a meaningful time period | n/a |
| Other analyses | | 17 | Report other analyses done—eg analyses of subgroups and interactions, and sensitivity analyses | 13-15 (Section 2) |
| Discussion | | | | |
| Key results | | 18 | Summarise key results with reference to study objectives | 15-19 (Section 2) |
| Limitations | | 19 | Discuss limitations of the study, taking into account sources of potential bias or imprecision. Discuss both direction and magnitude of any potential bias | 19 (Section 2) |
| Interpretation | | 20 | Give a cautious overall interpretation of results considering objectives, limitations, multiplicity of analyses, results from similar studies, and other relevant evidence | 15-20 (Section 2) |
| Generalisability | | 21 | Discuss the generalisability (external validity) of the study results | 19-20 (Section 2) |
| Other information | | | | |
| Funding | | 22 | Give the source of funding and the role of the funders for the present study and, if applicable, for the original study on which the present article is based | 21 (Section 2) |

*Give information separately for cases and controls in case-control studies and, if applicable, for exposed and unexposed groups in cohort and cross-sectional studies.

**Supplementary Table 2.** *Comparison of participants eligible at baseline (n=15,645), participants not included (n=13,519) and included in young adulthood (n=2,126), and comparison of fully observed variables between participants with complete data (n=1217) and incomplete data (n=1005) and participants in the weighted sample (n=7,815)*

|  | **Eligible at baseline** | **Not included in young adulthood** | **Included in young adulthood** |  |  |  | **Weighted sample** |
| --- | --- | --- | --- | --- | --- | --- | --- |
|  | **N=15,645)** | **N=13,519** | **N=2,126** | **Complete data (n=1121)** | **Incomplete data (n=1005)** | **Missingness (n=2,126)** | **N=7,815** |
|  | No. (%) | No. (%) | No. (%) | No. (%) | No. (%) | No. (%) |  |
| **Exposure** |  |  |  |  |  |  |  |
| Any ACE |  |  |  |  |  | 308 (14.49) |  |
| Yes | 3,819 (67.94) | 2,737 (71.97) | 1082 (59.52) | 623 (55.58) | 459 (65.85) |  | 4477 (57.29) |
| No | 1,802 (32.06) | 1,066 (28.03) | 736 (40.48) | 498 (44.42) | 238 (34.15) |  | 3338 (42.71) |
| Any Abuse |  |  |  |  |  | 366 (17.22) |  |
| Yes | 1,958 (30.37) | 1,385 (29.55) | 573 (32.56) | 353 (31.49) | 220 (34.43) |  | 2714 (34.74) |
| No | 4,489 (69.63) | 3,302 (70.45) | 1,187 (67.44) | 768 (68.51) | 419 (65.57) |  | 5100 (65.26) |
| Cumulative ACE |  |  |  |  |  | 483 (22.72) |  |
| 0 | 1,802 (44.02) | 1,066 (43.49) | 736 (44.80) | 498 (44.42) | 238 (45.59) |  | 3338 (42.71) |
| 1 | 1,411 (34.47) | 849 (34.64) | 562 (34.21) | 397 (35.41) | 165 (31.61) |  | 2592 (33.17) |
| 2 | 634 (15.49) | 376 (15.34) | 258 (15.70) | 166 (14.81) | 92 (17.62) |  | 1258 (16.10) |
| 3+ | 247 (6.03) | 160 (6.53) | 87 (5.30) | 60 (5.35) | 27 (5.17) |  | 627 (8.02) |
| Physical abuse |  |  |  |  |  | 360 (16.93) |  |
| Yes | 960 (14.90) | 634 (13.56) | 326 (18.46) | 207 (18.47) | 119 (18.45) |  | 1655 (21.18) |
| No | 5,481 (85.10) | 4,041 (86.44) | 1,440 (81.54) | 914 (81.53) | 526 (81.55) |  | 6160 (78.82) |
| Sexual abuse |  |  |  |  |  | 169 (7.95) |  |
| Yes | 254 (2.79) | 169 (2.36) | 85 (4.34) | 46 (4.10) | 39 (4.67) |  | 411 (5.26) |
| No | 8,859 (97.21) | 6,987 (97.64) | 1,872 (95.66) | 1075 (95.90) | 797 (95.33) |  | 7404 (94.74) |
| Emotional abuse |  |  |  |  |  | 360 (16.93) |  |
| Yes | 1,335 (19.31) | 1,008 (19.58) | 327 (18.52) | 207 (18.47) | 120 (18.60) |  | 1501 (19.20) |
| No | 5,580 (80.69) | 4,141 (80.42) | 1,439 (81.48) | 914 (81.53) | 525 (81.40) |  | 6314 (80.90) |
| Emotional neglect |  |  |  |  |  | 135 (6.35) |  |
| Yes | 1,105 (19.35) | 768 (20.65) | 337 (16.93) | 182 (16.24) | 155 (17.82) |  | 1555 (19.90) |
| No | 4,605 (80.65) | 2,951 (79.35) | 1,654 (83.07) | 939 (83.76) | 715 (82.18) |  | 6260 (80.10) |
| Bullying |  |  |  |  |  | 65 (3.06) |  |
| Yes | 1,705 (24.14) | 1,212 (24.23) | 493 (23.92) | 277 (24.71) | 216 (22.98) |  | 1989 (25.45) |
| No | 5,358 (75.86) | 3,790 (75.77) | 1,568 (76.08) | 844 (75.29) | 724 (77.02) |  | 5826 (74.55) |
| **Outcome** |  |  |  |  |  |  |  |
| Pooled Stage 1b+ outcome |  |  |  |  |  | 0 (0) |  |
| Presence | 118 (5.37) | 5 (7.14) | 113 (5.32) | 57 (5.08) | 56 (5.57) |  | 410 (5.24) |
| Absence | 2,078 (94.63) | 65 (92.86) | 2,013 (94.68) | 1064 (94.92) | 949 (94.43) |  | 7405 (94.76) |

**Supplementary Table 2.** *Comparison of participants eligible at baseline (n=15,645), participants not included (n=13,519) and included in young adulthood (n=2,126), and comparison of fully observed variables between participants with complete data (n=1217) and incomplete data (n=1005) and participants in the weighted sample (n=7,815; continued)*

|  | **Eligible at baseline** | **Not included in young adulthood** | **Included in young adulthood** | **Unweighted sample** | |  |  | **Weighted sample** |
| --- | --- | --- | --- | --- | --- | --- | --- | --- |
|  | **N=15,645)** | **N=13,519** | **N=2,126** | **Complete data (n=1121)** | **Incomplete data (n=1005)** | | **Missingness (n=2,126)** | **N=7,815** |
|  | No. (%) | No. (%) | No. (%) | No. (%) | No. (%) | | No. (%) |  |
| **Confounder** |  |  |  |  |  | |  |  |
| Child ethnicity |  |  |  |  |  | | 159 (7.48) |  |
| Non-white | 613 (5.05) | 544 (5.34) | 69 (3.51) | 35 (3.12) | 34 (4.02) | |  | 242 (3.10) |
| White | 11,537 (94.95) | 9,639 (94.66) | 1,898 (96.49) | 1086 (96.88) | 812 (95.98) | |  | 7573 (96.90) |
| Social class |  |  |  |  |  | | 179 (8.42) |  |
| Low | 6,699 (53.84) | 5,933 (56.53) | 766 (39.34) | 367 (32.74) | 399 (48.31) | |  | 3828 (48.99) |
| High | 5,744 (46.16) | 4,563 (43.47) | 1,181 (60.66) | 754 (67.26) | 427 (51.69) | |  | 3987 (51.01) |
| Maternal age at birth (years; mean [sd]) | 28.00 (4.96) | 27.70 (4.98) | 29.79 (4.44) | 30.18 (4.29) | 29.30 (4.57) | | 99 (4.66) | 28.86 (0.15) |
| Sex |  |  |  |  |  | | 0 (0) |  |
| Female | 7,219 (48.60) | 5,942 (46.69) | 1277 (60.07) | 670 (59.77) | 607 (60.40) | |  | 3950 (50.55) |
| Male | 7,634 (51.40) | 6,785 (53.31) | 849 (39.93) | 451 (40.23) | 398 (39.60) | |  | 3865 (49.45) |
| **Potential effect modifier** |  |  |  |  |  | |  |  |
| First-degree family history of mental disorders |  |  |  |  |  | | 378 (17.78) |  |
| Presence | 2,258 (23.56) | 1,940 (24.75) | 318 (18.19) | 166 (14.81) | 152 (24.24) | |  | 1640 (20.99) |
| Absence | 7,328 (76.44) | 5,898 (75.25) | 1,430 (81.81) | 955 (85.19) | 475 (75.76) | |  | 6175 (79.01) |
| Openness (mean [sd]) | 35.79 (5.65) | 35.45 (5.73) | 36.50 (5.42) | 36.82 (5.45) | 36.02 (5.32) | | 261 (12.28) | 36.23 (0.18) |
| Conscientiousness (mean [sd]) | 31.87 (5.82) | 31.63 (5.80) | 32.37 (5.82) | 32.34 (5.85) | 32.43 (5.78) | | 292 (13.73) | 32.29 (0.18) |
| Extraversion (mean [sd]) | 35.26 (6.88) | 35.20 (6.77) | 35.38 (7.08) | 35.32 (7.14) | 35.47 (7.01) | | 223 (10.49) | 35.27 (0.23) |
| Agreeableness (mean [sd]) | 37.87 (5.19) | 37.51 (5.22) | 38.62 (5.05) | 38.82 (5.04) | 38.33 (5.06) | | 255 (11.99) | 37.90 (0.18) |
| Neuroticism (mean [sd]) | 31.57 (6.57) | 31.30 (6.60) | 32.12 (6.48) | 32.45 (6.52) | 31.63 (6.40) | | 282 (13.26) | 32.21 (0.23) |
| Neurocognition (mean [sd]) | 103.97 (16.54) | 102.04 (16.47) | 109.36 (15.52) | 111.56 (15.19) | 106.37 (15.47) | | 184 (8.65) | 108.12 (0.51) |

**Supplementary Table 3.** *Categorisation of numerical effect modifiers for sensitivity and post-hoc analyses*

|  | **Low (1^st^ tertile)** | **Normal (2^nd^ tertile)** | **High (3^rd^ tertile)** |
| --- | --- | --- | --- |
| **Openness** | <34 | ≥34 and ≤39 | >39 |
| **Conscientiousness** | <30 | ≥30 and ≤35 | >35 |
| **Extraversion** | <33 | ≥33 and ≤39 | >39 |
| **Agreeableness** | <36 | ≥36 and ≤41 | >41 |
| **Emotional stability** | <29 | ≥29 and ≤35 | >35 |
|  | **Low (bottom quartile)** | **Normal (interquartile range)** | **High (top quartile)** |
| **Neurocognition** | <99 | ≥99 and ≤119 | >119 |

**Supplementary Table 4.** *Tetrachoric correlation between each exposure and each confounder*

|  | Sex | Ethnicity | Low social class | Maternal age^a^ |
| --- | --- | --- | --- | --- |
| Physical abuse | -0.1 | 0.1 | 0.0 | 0.2 |
| Sexual abuse | 0.3 | -0.1 | 0.1 | 0.2 |
| Emotional abuse | -0.1 | 0.1 | 0.0 | 0.3 |
| Emotional neglect | -0.1 | -0.1 | 0.1 | 0.1 |
| Bullying | -0.2 | -0.0 | 0.0 | 0.0 |

^a^ Pearson correlation was used for the correlation between each exposure and the continuous variable maternal age

**Supplementary Table 5.** *Multivariate logistic regression for the association between each ACE and each and the pooled stage 1b+ mental health outcome^a^*

|  | **Pooled stage 1b+ outcome** | | **Stage 1b+ depression** | | **Stage 1b+ psychosis** | | **Stage 1b+ anxiety** | |
| --- | --- | --- | --- | --- | --- | --- | --- | --- |
|  | Adjusted OR  (95% CI) | p-value | Adjusted OR  (95% CI) | p-value | Adjusted OR  (95% CI) | p-value | Adjusted OR  (95% CI) | p-value |
| Any ACE | 2.82 (1.71-4.65) | <.001 | 6.64 (2.63-16.77) | <.001 | 7.62 (1.79-32.49) | .006 | 2.67 (1.50-4.76) | .001 |
| Cumulative ACE |  |  |  |  |  |  |  |  |
| 1 | 1.74 (0.96-3.14) | .068 | 3.19 (1.12-9.11) | .030 | 4.63 (0.96-22.37) | .057 | 1.59 (0.79-3.18) | .190 |
| 2 | 4.01 (2.20-7.32) | <.001 | 10.31 (3.76-28.25) | <.001 | 10.24 (2.11-9.59) | .004 | 2.61 (1.79-7.26) | <.001 |
| 3+ | 3.63 (2.20-7.32) | .003 | 8.91 (2.53-31.44) | .001 | 13.11 (2.16-9.56) | .005 | 3.56 (1.34-9.43) | .011 |
| Physical abuse | 2.42 (1.54-3.80) | <.001 | 3.70 (2.03-6.75) | <.001 | 4.54 (1.95-10.56) | <.001 | 2.30 (1.36-3.89) | .002 |
| Sexual abuse | 2.24 (1.09-4.61) | .028 | 4.42 (2.01-9.72) | <.001 | 2.82 (0.83-9.55) | .096 | 2.46 (1.09-5.53) | .030 |
| Emotional abuse | 1.89 (1.17-3.03) | .009 | 2.76 (1.49-5.11) | .001 | 1.90 (0.73-4.99) | .191 | 2.23 (1.30-3.81) | .003 |
| Emotional neglect | 1.69 (1.06-2.70) | .029 | 1.71 (0.90-3.24) | .102 | 2.49 (1.06-5.86) | .037 | 1.70 (0.99-2.95) | .056 |
| Bullying | 2.01 (1.34-3.02) | .001 | 2.62 (1.52-4.53) | .001 | 2.80 (1.32-5.93) | .007 | 1.83 (1.13-2.97) | .014 |
| Any abuse | 2.07 (1.36-3.15) | .001 | 3.46 (1.91-6.29) | <.001 | 4.53 (1.84-11.18) | .001 | 2.07 (1.27-3.38) | .004 |

^a^ confounders maternal age, social class, ethnic group, and sex are adjusted for

**Sensitivity analysis**

**Supplementary Table 6.** *Prevalence of each variable and univariate and multivariate logistic regression for the association between each exposure and covariate and the stage 1b+ mental health outcome, analysis using the analytic sample*

|  |  | | **Analytic sample (N=2,126)** | | | | | |  |  | |  |  |  |  | **Weighted sample (N=7,815)^1^** | | |  |  |  | **Delta-adjusted sample (N= N=2,126)^2^** | | | |  |  |
| --- | --- | --- | --- | --- | --- | --- | --- | --- | --- | --- | --- | --- | --- | --- | --- | --- | --- | --- | --- | --- | --- | --- | --- | --- | --- | --- | --- |
|  | **Overall sample (n=2126)**  **(n/%)** | | | **Meeting Stage 1b+ outcome (n=113) (n/%)** | | | **Not meeting Stage 1b+ outcome (n=2013) (n/%)** | | | |  |  |  |  |  | |  |  | |  |  | |  |  |  | | |
|  | |  | | |  |  | | Unadjusted  OR (95% CI) | | | | p-value | Adjusted OR  (95% CI)^a^ | p-value | Unadjusted  OR (95% CI) | | p-value | Adjusted OR  (95% CI)^a^ | | p-value | Unadjusted  OR (95% CI) | | p-value | Adjusted OR  (95% CI)^a^ | p-value | | |
| ***Exposures*** | |  | | |  |  | |  | | | |  |  |  |  | |  |  | |  |  | |  |  |  | | |
| Any ACE | | 1082 (59.52) | | | 79 (79.80) | 1003 (58.35) | | 2.82 (1.71-4.65) | | | | <.001 | 2.96 (1.76-4.97) | <.001 | 3.67 (1.96-6.87) | | <.001 | 3.81 (2.04-7.10) | | <.001 | 2.64 (1.66-4.20) | | <.001 | 2.72 (1.70-4.37) | <.001 | | |
| Cumulative ACE | |  | | |  |  | |  | | | |  |  |  |  | |  |  | |  |  | |  |  |  | | |
| 1 | | 562 (34.21) | | | 26 (32.50) | 536 (34.29) | | 1.74 (0.96-3.14) | | | | .068 | 1.81 (0.98-3.32) | .058 | 2.22 (0.98-5.03) | | .055 | 2.33 (1.02-5.31) | | .044 | 1.81 (1.03- 3.17) | | <.001 | 1.87 (1.06-3.31) | .031 | | |
| 2 | | 258 (15.70) | | | 26 (32.50) | 232 (14.84) | | 4.01 (2.20-7.32) | | | | <.001 | 4.08 (2.18-7.65) | <.001 | 5.47 (2.50-11.97) | | <.001 | 5.82 (2.63-12.88) | | <.001 | 3.88 (2.24- 6.71) | | <.001 | 4.01 (2.29-7.04) | <.001 | | |
| 3+ | | 87 (5.30) | | | 8 (10.00) | 79 (5.05) | | 3.63 (2.20-7.32) | | | | .003 | 3.89 (1.64-9.22) | .002 | 6.22 (2.31-16.74) | | <.001 | 6.00 (2.23-16.16) | | <.001 | 4.43 (2.10- 9.31) | | <.001 | 4.57 (2.16-9.66) | <.001 | | |
| Physical abuse | | 326 (18.46) | | | 31 (34.07) | 295 (17.61) | | 2.42 (1.54-3.80) | | | | <.001 | 2.54 (1.60-4.03) | <.001 | 3.07 (1.57-5.99) | | .001 | 3.10 (1.56-6.19) | | .001 | 2.48 (1.62-3.80) | | <.001 | 2.51 (1.64-3.85) | <.001 | | |
| Sexual abuse | | 85 (4.34) | | | 9 (8.74) | 76 (4.10) | | 2.24 (1.09-4.61) | | | | .028 | 1.87 (0.87-4.02) | .111 | 1.85 (0.63-5.44) | | .267 | 1.57 (0.53-4.64) | | .412 | 2.09 (1.02-4.26) | | .044 | 1.83 (0.88-3.79) | .105 | | |
| Emotional abuse | | 327 (18.52) | | | 26 (29.21) | 301 (17.95) | | 1.89 (1.17-3.03) | | | | .009 | 1.94 (1.19-3.16) | .008 | 2.04 (0.98-4.26) | | .058 | 2.06 (0.99-4.29) | | .052 | 1.91 (1.19-3.08) | | .008 | 1.88 (1.16-3.05) | .011 | | |
| Emotional neglect | | 337 (16.93) | | | 25 (25.00) | 312 (16.50) | | 1.69 (1.06-2.70) | | | | .029 | 1.80 (1.10-2.96) | .020 | 1.81 (0.93-3.53) | | .080 | 1.81 (0.91-3.59) | | .092 | 1.59 (0.99-2.56) | | .056 | 1.66 (1.03-2.67) | .039 | | |
| Bullying | | 493 (23.92) | | | 40 (37.74) | 453 (23.17) | | 2.01 (1.34-3.02) | | | | .001 | 2.02 (1.31-3.12) | .002 | 2.72 (1.48-5.00) | | .001 | 2.84 (1.54-5.23) | | .001 | 2.06 (1.38-3.07) | | <.001 | 2.21 (1.47-3.32) | <.001 | | |
| Any abuse | | 573 (32.56) | | | 45 (48.91) | 528 (31.65) | | 2.07 (1.36-3.15) | | | | .001 | 2.10 (1.36-3.24) | .001 | 2.55 (1.32-4.92) | | .005 | 2.55 (1.32-4.93) | | .006 | 2.08 (1.38-3.13) | | <.001 | 2.05 (1.35-3.10) | .001 | | |

^a^ confounders include maternal age, social class, ethnic group, and sex; ^1^ sensitivity analysis using IPW/MI; ^2^ sensitivity analysis using MI, with delta value of 0.2 to inform the weights, corresponding to the Stage 1b+ outcome increasing the odds of having complete data for any ACE by 1.25-fold

**Supplementary Table 6.** *Prevalence of each variable and univariate and multivariate logistic regression for the association between each exposure and covariate and the stage 1b+ mental health outcome, analysis using the analytic sample (continued)*

|  |  | | **Analytic sample (N=2,126)** | | | | |  |  |  |  |  |  | **Weighted sample (N=7,815)^1^** | | |  |  |  | **Delta-adjusted sample (N= N=2,126)^2^** | | | |  |  |
| --- | --- | --- | --- | --- | --- | --- | --- | --- | --- | --- | --- | --- | --- | --- | --- | --- | --- | --- | --- | --- | --- | --- | --- | --- | --- |
|  | **Overall sample (n=2126)**  **(n/%)** | | | **Stage 1b+ outcome (n=113, n/%)** | | **Not meeting stage 1b+ outcome (n=2013, n/%)** | | |  |  |  |  |  | |  |  | |  |  | |  |  |  | | |
|  | |  | | |  |  | Unadjusted  OR (95% CI) | | | p-value | Adjusted OR  (95% CI)^a^ | p-value | Unadjusted  OR (95% CI) | | p-value | Adjusted OR  (95% CI)^a^ | | p-value | Unadjusted  OR (95% CI) | | p-value | Adjusted OR  (95% CI)^a^ | p-value | | |
| **Covariates** | |  | | |  |  |  | | |  |  |  |  | |  |  | |  |  | |  |  |  | | |
| Low social class | | 766 (39.34) | | | 43 (42.57) | 723 (39.17) | 1.15 (0.77-1.73) | | | .495 | 1.14 (0.72-1.80) | .589 | 1.31 (0.75-2.32) | | .341 | 1.26 (0.70-2.25) | | .444 | 1.19 (0.80-1.76) | | .389 | 1.17 (0.77-1.77) | .468 | | |
| Ethnicity (non-white) | | 69 (3.51) | | | 6 (5.77) | 63 (3.38) | 1.75 (0.74-4.14) | | | .203 | 1.66 (0.58-4.78) | .349 | 0.61 (0.75-2.32) | | .512 | 0.60 (0.14-2.60) | | .496 | 1.66 (0.69-4.01) | | .258 | 1.78 (0.70-4.04) | .247 | | |
| First-degree family history of mental disorders | | 318 (18.19) | | | 32 (32.32) | 286 (17.34) | 2.28 (1.47-3.53) | | | <.001 | 2.27 (1.42-3.61) | <.001 | 2.06 (1.05-4.06) | | .036 | 1.96 (1.02-3.77) | | .044 | 2.21 (1.43-3.41) | | <.001 | 2.13 (1.37) | .001 | | |
| Sex (female) | | 1277 (60.07) | | | 83 (73.45) | 1194 (59.31) | 1.90 (1.24-2.91) | | | .003 | 1.89 (1.17-3.06) | .009 | 1.62 (0.85-3.08) | | .139 | 1.60 (0.84-3.06) | | .152 | 1.90 (1.24-2.91) | | .003 | 1.88 (1.23-2.90) | .004 | | |

^a^ confounders include maternal age, social class, ethnic group, and sex; ^1^ sensitivity analysis using IPW/MI; ^2^ sensitivity analysis using MI, with delta value of 0.2 to inform the weights, corresponding to the Stage 1b+ outcome increasing the odds of having complete data for any ACE by 1.25-fold

**Supplementary Table 6.** *Prevalence of each variable and univariate and multivariate logistic regression for the association between each exposure and covariate and the stage 1b+ mental health outcome, analysis using the analytic sample (continued)*

|  |  | | **Analytic sample (N=2,126)** | | | | |  |  |  |  |  |  | **Weighted sample (N=7,815)^1^** | | |  |  |  | **Delta-adjusted sample (N= N=2,126)^2^** | | | |  |  |
| --- | --- | --- | --- | --- | --- | --- | --- | --- | --- | --- | --- | --- | --- | --- | --- | --- | --- | --- | --- | --- | --- | --- | --- | --- | --- |
|  | **Overall sample (n=2126)**  **(n/%)** | | | **Stage 1b+ outcome (n=113, n/%)** | | **Not meeting stage 1b+ outcome (n=2013, n/%)** | | |  |  |  |  |  | |  |  | |  |  | |  |  |  | | |
|  | |  | | |  |  | Unadjusted  OR (95% CI) | | | p-value | Adjusted OR  (95% CI)^a^ | p-value | Unadjusted  OR (95% CI) | | p-value | Adjusted OR  (95% CI)^a^ | | p-value | Unadjusted  OR (95% CI) | | p-value | Adjusted OR  (95% CI)^a^ | p-value | | |
| ***Covariates*** | |  | | |  |  |  | | |  |  |  |  | |  |  | |  |  | |  |  |  | | |
| Maternal age (years) | | 29.79 (4.44) | | | 29.72 (4.44) | 29.79 (4.44) | 1.00 (0.95-1.04) | | | .870 | 1.00 (0.96-1.06) | .860 | 0.98 (0.93-1.05) | | .623 | 1.00 (0.94-1.06) | | .882 | 1.00 (0.95-1.04) | | .880 | 1.00 (0.96-1.05) | .848 | | |
| Personality (openness) | | 36.50 (5.42) | | | 37.90 (6.90) | 36.43 (5.32) | 1.05 (1.01-1.09) | | | .010 | 1.06 (1.02-1.10) | .007 | 1.08 (1.00-1.16) | | .064 | 1.09 (1.01-1.18) | | .031 | 1.05 (1.01-1.10) | | .026 | 1.06 (1.01-1.11) | .014 | | |
| Personality (conscientiousness) | | 32.37 (5.82) | | | 29.95 (6.01) | 32.50 (5.78) | 0.93 (0.89-0.96) | | | <.001 | 0.92 (0.89-0.95) | <.001 | 0.96 (0.91-1.01) | | .097 | 0.96 (0.91-1.01) | | .136 | 0.93 (0.89-0.96) | | <.001 | 0.93 (0.87-0.97) | <.001 | | |
| Personality (extraversion) | | 35.38 (7.08) | | | 34.96 (8.71) | 35.41 (6.99) | 0.99 (0.96-1.02) | | | .549 | 1.00 (0.97-1.03) | .932 | 1.02 (0.96-1.07) | | .581 | 1.01 (0.96-1.07) | | .695 | 0.99 (0.96-1.03) | | .694 | 0.99 (0.96-1.02) | .459 | | |
| Personality (agreeableness) | | 38.62 (5.05) | | | 39.90 (5.76) | 38.55 (5.00) | 1.06 (1.01-1.10) | | | .011 | 1.05 (1.00-1.10) | .042 | 1.06 (0.99-1.14) | | .083 | 1.06 (0.98-1.14) | | .153 | 1.06 (1.01-1.11) | | .011 | 1.05 (1.00-1.10) | .047 | | |
| Personality (emotional stability) | | 32.12 (6.48) | | | 27.16 (7.73) | 32.39 (6.30) | 0.88 (0.85-0.91) | | | <.001 | 0.89 (0.86-0.92) | <.001 | 0.89 (0.85-0.94) | | <.001 | 0.89 (0.85-0.94) | | <.001 | 0.88 (0.85-0.92) | | <.001 | 0.89 (0.85-0.92) | <.001 | | |
| Neurocognition | | 109.36 (15.52) | | | 106.87 (15.39) | 109.50 (15.52) | 0.99 (0.98-1.00) | | | .096 | 0.99 (0.98-1.00) | .193 | 0.99 (0.97-1.01) | | .205 | 0.99 (0.97-1.01) | | .351 | 0.99 (0.98-1.00) | | .104 | 0.99 (0.98-1.00) | .177 | | |

^a^ confounders include maternal age, social class, ethnic group, and sex; ^1^ sensitivity analysis using IPW/MI; ^2^ sensitivity analysis using MI, with delta value of 0.2 to inform the weights, corresponding to the Stage 1b+ outcome increasing the odds of having complete data for any ACE by 1.25-fold

**Supplementary Table 7.** *Logistic regression models between the exposures and the outcome across levels of each effect modifier, with F-test for interaction, complete case analysis*

|  | **Complete case sample (N=2,126)** |  |  | **Weighted sample (N=7,815)^1^** |  | **Delta-adjusted sample (N= N=2,126)^2^** | |  |
| --- | --- | --- | --- | --- | --- | --- | --- | --- |
| **Outcome** | **Exposure** | **OR (95% CI)^a^** | **F-test p-value** | **OR (95% CI)^a^** | **F-test p-value** | **OR (95% CI)^a^** | **F-test p-value** | |
| **Stage 1b+** | **Any abuse** |  |  |  |  |  |  | |
|  | Sex^b^ |  | .591 |  | NA |  | NA | |
|  | Female | 2.26 (1.36-3.76) |  | NA |  | NA |  | |
|  | Male |  |  | NA |  | NA |  | |
|  | Family history of mental disorder |  | .672 |  | NA |  | NA | |
|  | Present | 1.86 (0.78-4.44) |  | NA |  | NA |  | |
|  | Absence |  |  | NA |  | NA |  | |
|  | Openness | 1.02 (0.93-1.11) | .737 | NA | NA | NA | NA | |
|  | Conscientiousness | 0.98 (0.90-1.06) | .635 | NA | NA | NA | NA | |
|  | Extraversion | 0.96 (0.87-1.05) | .393 | NA | NA | NA | NA | |
|  | Agreeableness | 0.96 (0.87-1.05) | .346 | NA | NA | NA | NA | |
|  | Emotional stability | 0.94 (0.87-1.01) | .091 | NA | NA | NA | NA | |
|  | Neurocognition | 0.99 (0.96-1.02) | .508 | NA | NA | NA | NA | |
|  | **Emotional neglect** |  |  |  |  |  |  | |
|  | Sex^b^ |  | **.021** |  | **.047** |  | **.023** | |
|  | Female | 1.14 (0.58-2.24) |  | 0.90 (0.34-2.35) |  | 1.11 (0.58-2.12) |  | |
|  | Male |  |  | **4.04 (1.27-12.87)** |  | **3.45 (1.62-7.45)** |  | |
|  | Family history of mental disorder |  |  |  |  |  |  | |
|  | Present | 1.78 (0.69-4.56) | .883 | NA | NA | NA | NA | |
|  | Absence |  |  | NA | NA | NA | NA | |
|  | Openness | 0.83 (0.75-0.91) | **<.001** | 0.92 (0.77-1.11) | .385 | **0.84 (0.76-0.94)** | **.002** | |
|  | Conscientiousness | 1.01 (0.92-1.11) | .844 | NA | NA | NA | NA | |
|  | Extraversion | 0.89 (0.80-0.99) | **.001** | **0.88 (0.80-0.98)** | **.016** | **0.90 (0.84-0.97)** | **.047** | |
|  | Agreeableness | 0.89 (0.80-0.99) | .026 | NA | NA | NA | NA | |
|  | Emotional stability | 1.03 (0.94-1.12) | .532 | NA | NA | NA | NA | |
|  | Neurocognition | 0.98 (0.95-1.01) | .152 | NA | NA | NA | NA | |
|  | **Bullying** |  |  |  |  |  |  | |
|  | Sex^b^ |  | .871 |  | NA |  |  | |
|  | Female | 2.07 (1.23-3.48)^c^ |  | NA |  | NA | NA | |
|  | Male |  |  | NA |  | NA | NA | |
|  | Family history of mental disorder (present) |  | .302 |  |  |  |  | |
|  | Present | 2.92 (1.29-6.60)^c^ |  | NA | NA | NA | NA | |
|  | Absence |  |  | NA | NA | NA | NA | |
|  | Openness | 0.90 (0.83-0.98) | **.018** | NA | NA | NA | NA | |
|  | Conscientiousness | 0.97 (0.89-1.05) | .470 | NA | NA | NA | NA | |
|  | Extraversion | 0.98 (0.90-1.08) | .251 | NA | NA | NA | NA | |
|  | Agreeableness | 0.98 (0.90-1.08) | .749 | NA | NA | NA | NA | |
|  | Emotional stability | 0.97 (0.90-1.05) | .493 | NA | NA | NA | NA | |
|  | Neurocognition | 0.98 (0.96-1.01) | .225 | NA | NA | NA | NA | |

^a^ confounders maternal age, social class, ethnic group, and sex are adjusted for; effect modifiers sex and family history of mental disorder are binary, while effect modifiers openness, conscientiousness, extraversion, agreeableness, emotional stability and neurocognition are numerical; ^b^ confounders maternal age, social class and ethnic group are adjusted for; ^1^ sensitivity analysis using IPW/MI; ^2^ sensitivity analysis using MI, with delta value of 0.2 to inform the weights, corresponding to the Stage 1b+ outcome increasing the odds of having complete data for any ACE by 1.25-fold

**Supplementary Table 8a.** *Logistic regression models between the exposures and the outcome across levels of the dichotomous effect modifiers, with ROR and RERI, complete case analysis*

|  |  | |  |  | **Sex^a^** |  |  |
| --- | --- | --- | --- | --- | --- | --- | --- |
|  | Female | |  |  |  | Male |  |
|  | OR (95% CI) | | p-value | ROR | RERI | OR (95% CI) | p-value |
| **Complete case sample (N=2,126)** |  | |  |  |  |  |  |
| **Emotional neglect** | 1.14 (0.58-2.24) | | .702 | **0.29 (0.10-0.84)^b^** | -2.53 (-6.01-0.94) | 3.87 (1.75-8.59) | .001 |
| **Weighted sample (N=7,815)^1^** |  | |  |  |  |  |  |
| **Emotional neglect** | 0.90 (0.34-2.35) | | .830 | **0.22 (0.05-0.98)^b^** | -3.29 (-8.62-2.04) | 4.04 (1.27-12.87) | .018 |
| **Delta-adjusted sample (N= N=2,126)^2^** | |  |  |  |  |  |  |
| **Emotional neglect** | 1.11 (0.58-2.12) | | .762 | **0.32 (0.12-0.86)^b^** | -2.15 (-5.29-0.98) | 3.45 (1.62-7.45) | .001 |

^a^ confounders maternal age, social class and ethnic group are adjusted for; ^b^ p-value<.05; ^1^ sensitivity analysis using IPW/MI; ^2^ sensitivity analysis using MI, with delta value of 0.2 to inform the weights, corresponding to the Stage 1b+ outcome increasing the odds of having complete data for any ACE by 1.25-fold

**Supplementary Table 8b.***Logistic regression models^a^ between the exposures and the outcome across levels of the trichotomous effect modifiers, with ROR and RERI, complete case analysis*

|  | | | | | | | | | | | | | | | | **Openness^a^** | | | | | |
| --- | --- | --- | --- | --- | --- | --- | --- | --- | --- | --- | --- | --- | --- | --- | --- | --- | --- | --- | --- | --- | --- |
|  | Low openness (1^st^ tertile) | | | | | | | | | | | | | | | Normal openness (2^nd^ tertile) | | High openness (3^rd^ tertile) | | | |
|  | OR (95% CI) | p-value | | | | ROR | | | | | | | RERI | | | OR (95% CI) | p-value | OR (95% CI) | p-value | ROR | RERI |
| **Analytic sample (N=2,126)** | | |  | |  |  | | | | | | |  | | |  |  |  |  |  |  |
| **Emotional neglect** | 5.82 (2.31-14.69) | <.001 | | | | 2.99 (0.79-11.26) | | | | | | | **2.64 (0.24-5.04)^b^** | | | 1.97 (0.76-5.10) | .165 | 0.67 (0.23-1.97) | .472 | 0.34 (0.08-1.42) | -3.48 (-7.02-0.06) |
| **Weighted sample (N=7,815)^1^** | | | |  | | |  | |  | | | |  | | |  |  |  |  |  |  |
| **Emotional neglect** | 2.68 (0.75-9.56) | .130 | | | | 1.14 (0.20-6.41) | | | | | | | -0.11 (-1.96-1.75) | | | 2.34 (0.73-7.52) | .153 | 1.47 (0.44-4.88) | .532 | 0.63 (0.12-3.28) | -0.39 (-4.69-3.91) |
| **Delta-adjusted sample (N= N=2,126)^2^** | | | | | | | |  | |  |  | |  | | |  |  |  |  |  |  |
| **Emotional neglect** | 4.49 (1.95-10.32) | <.001 | | | | 2.81 (0.77-10.20) | | | | | | | **2.04 (0.18-3.89)^b^** | | | 1.60 (0.62-4.09) | .329 | 0.68 (0.25-1.86) | .457 | 0.42 (0.11-1.66) | -2.61 ( -5.29-0.07) |
|  | | | | | | | | | | | | | | | | **Extraversion^a^** | | | | | |
|  | Low extraversion (1^st^ tertile) | | | | | | | | | | | | | | | Normal extraversion (2^nd^ tertile) | | High extraversion (3^rd^ tertile) | | | |
|  | OR (95% CI) | p-value | | | | ROR | | | | | | | RERI | | | OR (95% CI) | p-value | OR (95% CI) | p-value | ROR | RERI |
| **Analytic sample (N=2,126)** | | |  | |  |  | | | | | | |  | | |  |  |  |  |  |  |
| Emotional neglect | 2.88 (1.25-6.59) | .013 | | | | 1.13 (0.35-3.63) | | | | | | | 1.46 (-0.39-3.31) | | | 2.31 (1.01-5.28) | .046 | NA | NA | NA | NA |
| **Weighted sample (N=7,815)^1^** | | | |  | | | |  | | |  | |  | | |  |  |  |  |  |  |
| **Emotional neglect** | 2.97 (1.06- 8.31) | .038 | | | | NA | | | | | | | 1.93 (-1.71-5.57) | | | 1.24 (0.42-3.71) | .698 | NA | NA | NA | NA |
| **Delta-adjusted sample (N= N=2,126)^2^** | | | | | | | |  | | | |  | |  |  |  |  |  |  |  |  |
| **Emotional neglect** | 2.67 (1.30- 5.49) | .008 | | | | NA | | | | | | | 1.47 ( -0.41-3.36) | | | 1.15 (0.57-2.32) | .704 | NA | NA | NA | NA |

^a^ confounders maternal age, social class, ethnic group, and sex are adjusted for; ^b^ p-value <.05; ^1^ sensitivity analysis using IPW/MI; ^2^ sensitivity analysis using MI, with delta value of 0.2 to inform the weights, corresponding to the Stage 1b+ outcome increasing the odds of having complete data for any ACE by 1.25-fold; NA no applicable as model did not converge due to low cell numbers

**Supplementary Table 9.** *Univariate and multivariable logistic regression models^a^ for the association between each exposure and the pooled outcome (excluding each stage 1b+ outcome)*

| **Depression/psychosis** |  |  |  |  |
| --- | --- | --- | --- | --- |
|  | Crude OR (95% CI) | p-value | Adjusted OR (95% CI) | p-value |
| Any ACE | 6.11 (2.78-13.45) | <.001 | 7.04 (3.01-16.50) | <.001 |
| Cumulative ACE |  |  |  |  |
| 1 | 3.05 (1.25-7.47) | .015 | 3.43 (1.32-8.93) | .011 |
| 2 | 9.71 (4.10-23.01) | <.001 | 10.88 (4.28-27.62) | <.001 |
| 3+ | 9.11 (3.12-26.64) | <.001 | 10.77 (3.52-32.94) | <.001 |
| Physical abuse | 3.58 (2.12-6.06) | <.001 | 3.90 (2.26-6.72) | <.001 |
| Sexual abuse | 3.08 (1.43-6.67) | .004 | 3.63 (1.15-6.02) | .022 |
| Emotional abuse | 2.40 (1.38-4.19) | .002 | 2.54 (1.43-4.51) | .002 |
| Emotional neglect | 1.85 (1.06-3.21) | .030 | 2.09 (1.17-3.73) | .013 |
| Bullying | 2.81 (1.75-4.52) | <.001 | 2.75 (1.65-4.59) | <.001 |
| Any abuse | 3.35 (1.98-5.65) | <.001 | 3.54 (2.05-6.10) | <.001 |
| **Depression/anxiety** |  |  |  |  |
|  | Crude OR (95% CI) | p-value | Adjusted OR (95% CI) | p-value |
| Any ACE | 2.72 (1.60-4.60) | <.001 | 2.89 (1.67-5.01) | <.001 |
| Cumulative ACE |  |  |  |  |
| 1 | 1.63 (0.86-3.06) | .133 | 1.78 (0.93-3.39) | .082 |
| 2 | 3.72 (1.96-7.05) | <.001 | 3.71 (1.89-7.29) | <.001 |
| 3+ | 3.49 (1.41-8.61) | .007 | 3.78 (1.51-9.44) | .004 |
| Physical abuse | 2.36 (1.46-3.81) | <.001 | 2.43 (1.49-3.99) | <.001 |
| Sexual abuse | 2.65 (1.28-5.48) | .008 | 2.12 (0.98-4.59) | .057 |
| Emotional abuse | 2.16 (1.32-3.54) | .002 | 2.20 (1.33-3.66) | .002 |
| Emotional neglect | 1.47 (0.88-2.46) | .140 | 1.53 (0.89-2.65) | .125 |
| Bullying | 1.83 (1.18-2.85) | .007 | 1.90 (1.19-3.04) | .008 |
| Any abuse | 2.10 (1.34-3.28) | .001 | 2.07 (1.31-3.27) | .002 |
| **Psychosis/anxiety** |  |  |  |  |
|  | Crude OR (95% CI) | p-value | Adjusted OR (95% CI) | p-value |
| Any ACE | 2.84 (1.65-4.87) | <.001 | 3.06 (1.75-5.36) | <.001 |
| Cumulative ACE |  |  |  |  |
| 1 | 1.72 (0.91-3.28) | .097 | 1.81 (0.93-3.50) | .080 |
| 2 | 3.94 (2.06-7.55) | <.001 | 4.22 (2.14-8.29) | <.001 |
| 3+ | 3.70 (1.49-9.19) | .005 | 3.98 (1.58-10.00) | .003 |
| Physical abuse | 2.36 (1.45-3.85) | .001 | 2.60 (1.58-4.27) | <.001 |
| Sexual abuse | 1.98 (0.89-4.44) | .095 | 1.57 (0.66-3.75) | .308 |
| Emotional abuse | 1.89 (1.13-3.15) | .015 | 2.06 (1.22-3.47) | .007 |
| Emotional neglect | 2.04 (1.25-3.32) | .004 | 2.10 (1.25-3.52) | .005 |
| Bullying | 2.10 (1.36-3.25) | .001 | 2.09 (1.32-3.33) | .002 |
| Any abuse | 2.04 (1.29-3.21) | .002 | 2.13 (1.34-3.40) | .001 |

^a^ confounders maternal age, social class, ethnic group, and sex are adjusted for

**Supplementary Table 10a.** *Logistic regression models between emotional neglect^a^ and the pooled outcome (excluding each stage 1b+ outcome) across levels of the dichotomous effect modifiers, with ROR and RERI*

|  |  |  |  | **Sex** | |  |  |
| --- | --- | --- | --- | --- | --- | --- | --- |
|  | Female |  |  | |  | Male |  |
| **Depression/psychosis** |  |  |  | |  |  |  |
|  | OR (95% CI) | p-value | ROR | | RERI | OR (95% CI) | p-value |
| Emotional neglect | 1.16 (0.50-2.67) | .733 | 0.23 (0.07-0.80)^b^ | | -3.63 (-8.59-1.32) | 5.00 (1.99-12.57) | .001 |
| **Depression/anxiety** | Female |  |  | |  | Male |  |
|  | OR (95% CI) | p-value | ROR | | RERI | OR (95% CI) | p-value |
| Emotional neglect | 1.11 (0.55-2.26) | .763 | 0.38 (0.12-1.21) | | -1.62 (-4.86-1.62) | 2.91 (1.17-7.29) | .022 |
| **Psychosis/anxiety** | Female |  |  | |  | Male |  |
|  | OR (95% CI) | p-value | ROR | | RERI | OR (95% CI) | p-value |
| Emotional neglect | 1.21 (0.60-2.47) | .592 | 0.22 (0.07-0.68)^b^ | | -3.84 (-8.85-1.17) | 5.44 (2.30-12.90) | <.001 |

^a^ statistically significant evidence was available only for the interaction with emotional neglect

**Supplementary Table 10b.** *Logistic regression models^a^ between the exposures and the outcome across levels of the trichotomous effect modifiers, with F-test for interaction, ROR and RERI*

|  | | | | | | | | | **Openness^b^** | | | | | | | | | | | |
| --- | --- | --- | --- | --- | --- | --- | --- | --- | --- | --- | --- | --- | --- | --- | --- | --- | --- | --- | --- | --- |
|  | Low openness (1^st^ tertile) | | | | | | | | Normal openness (2^nd^ tertile) | | | High openness (3^rd^ tertile) | | | | | | | | |
| **Depression/psychosis** |  | | | | | | | |  | | |  | | | | | | | | |
|  | OR (95% CI) | p-value | ROR | | | | | RERI | OR (95% CI) | p-value | | OR (95% CI) | p-value | ROR | | | | | RERI | |
| Emotional neglect | 4.19 (1.48-11.87) | .007 | 1.57 (0.35-7.02) | | | | | 2.04 (-0.98-5.05) | 2.71 (0.92-8.01) | .071 | | 1.05 (0.35-3.14) | .930 | 0.39 (0.08-1.79) | | | | | -2.22 (-6.72-2.28) | |
| **Depression/anxiety** | Low openness (1^st^ tertile) | | | | | | | | Normal openness (2^nd^ tertile) | | | High openness (3^rd^ tertile) | | | | | | | | |
|  | OR (95% CI) | p-value | ROR | | | | | RERI | OR (95% CI) | p-value | | OR (95% CI) | p-value | ROR | | | | | RERI | |
| Emotional neglect | 4.89 (1.77-13.53) | .002 | 2.10 (0.51-8.57) | | | | | 2.14 (-0.03-4.30) | 2.35 (0.88-6.26) | .087 | | 0.35 (0.08-1.50) | .158 | 0.15 (0.03-0.84)^c^ | | | | | -4.72 (-8.47--0.96)^c^ | |
| **Psychosis/anxiety** | Low openness (1^st^ tertile) | | | | | | | | Normal openness (2^nd^ tertile) | | | High openness (3^rd^ tertile) | | | | | | | | |
|  | OR (95% CI) | p-value | ROR | | | | | RERI | OR (95% CI) | p-value | | OR (95% CI) | p-value | ROR | | | | | RERI | |
| Emotional neglect | 9.38 (3.21-27.37) | <.001 | 5.25 (1.19-23.19)^c^ | | | | | 3.17 (0.38-5.97)^c^ | 1.79 (0.64-5.04) | .267 | | 0.85 (0.29-2.52) | .771 | 0.47 (0.10-2.08) | | | | | -3.42 (-7.58-0.74) | |
|  | | | | | | | | | **Extraversion^b^** | | | | | | | | | | | |
| **Depression/psychosis** | Low extraversion (1^st^ tertile) | | | | | | | | Normal extraversion (2^nd^ tertile) | | | High extraversion (3^rd^ tertile) | | | | | | | | |
|  | OR (95% CI) | p-value | ROR | | | | | RERI | OR (95% CI) | p-value | | OR (95% CI) | p-value | ROR | | | | | RERI | |
| Emotional neglect | 1.41 (1.39-8.42) | .008 | 1.16 (0.39-6.28) | | | | | 2.49 (-0.33-5.32) | 2.00 (0.69-5.82) | .201 | | NA | NA | NA | | | | | NA | |
| **Depression/anxiety** | Low extraversion (1^st^ tertile) | | | |  |  | |  | Normal extraversion (2^nd^ tertile) | |  | High extraversion (3^rd^ tertile) | | | |  |  | | |  |
|  | OR (95% CI) | p-value | ROR | | | | | RERI | OR (95% CI) | p-value | | OR (95% CI) | p-value | ROR | | | | | RERI | |
| Emotional neglect | 2.45 (1.00-6.02) | 0.050 | 1.18 (0.33-4.20) | | | | | 1.30 (-0.48-3.09) | 1.84 (0.74-4.53) | .187 | | NA | NA | NA | | | | | NA | |
| **Psychosis/anxiety** | Low extraversion (1^st^ tertile) | | |  | | |  |  | Normal extraversion (2^nd^ tertile) | |  | High extraversion (3^rd^ tertile) | | |  | | |  |  | |
|  | OR (95% CI) | p-value | ROR | | | | | RERI | OR (95% CI) | p-value | | OR (95% CI) | p-value | ROR | | | | | RERI | |
| Emotional neglect | 3.49 (1.47-8.28) | .005 | 1.08 (0.32-3.63) | | | | | 1.70 ( -0.56-3.97) | 2.93 (1.24-6.91) | .014 | | NA | NA | NA | | | | | NA | |

^a^ confounders maternal age, social class, ethnic group, and sex are adjusted for; ^b^ statistically significant evidence was available only for the interaction with emotional neglect ^c^ p-value for F-test <.05; NA not applicable as model did not converge due to missingness in cell
