# Supplementary material for: Adverse childhood experiences and their differential relationships with transdiagnostic mental health outcomes in young adults
Source: Psychol Med. 2025 May 22;55:e147. doi: 10.1017/S0033291725000893 (PMC12115273; doi:10.1017/S0033291725000893)
Supplement: Chen et al. supplementary material 2 — Chen et al. supplementary material [file S0033291725000893sup002.docx]

**Supplemental Figures**

**Supplementary Figure 1.** *Flow chart for the number of participants in each wave*

Pregnancies enrolled in the ALSPAC cohort

**(n=14541)**

Participants joined during adolescence & young adulthood

**(n=1658)**

Participants with complete data for the exposure any abuse, emotional neglect, or bullying, and the pooled stage 1b mental health outcome

**(n=2126)**

Participants with complete data for the exposure: any abuse, emotional neglect, or bullying,

**(n=8682)**

Total eligible sample in the ALSPAC cohort

**(n=15645)**

Eligible infant participants in the ALSPAC cohort

**(n=13988)**

**Supplementary Figure 2*.*** *Categorising the 19 types of ACE into external threat induced by abuse and neglect, household factors, and socio-economic factors*

***Exposure***

Physical abuse

Sexual abuse

Emotional abuse

Emotional neglect

Bullying

Personal adversities (maltreatment)

Household adversities

Household violence

Household substance use problems

Parental mental health problems

Parental convictions

Parental separation

Violence between child and partner

Physical illness of child

Physical illness of parent

Poor parent-child bond

19 time-collapsed ACEs

Social class

Financial difficulties

Neighbourhood satisfaction

Social support revived by parent

Social support received by child

Socio-economic adversities

**Supplementary Figure 3.** *The Directed Acyclic Graph (DAG) for the association between ACEs and stage 1b+ mental health outcome*

Agreeableness (13.5 years old)

Neuroticism (13.5 years old)

Low social class

Maternal age

Sex

Ethnicity

Neurocognition (8 years old)

Sex

Parental history of mental disorders (pregnancy to 8 years old)

Extraversion (13.5 years old)

Conscientiousness (13.5 years old)

Openness (13.5 years old)

Stage 1b+ mental health outcomes

ACEs (0-16 years old)
