# Supplementary material for: Adverse childhood experiences and their differential relationships with transdiagnostic mental health outcomes in young adults
Source: Psychol Med. 2025 May 22;55:e147. doi: 10.1017/S0033291725000893 (PMC12115273; doi:10.1017/S0033291725000893)
Supplement: Chen et al. supplementary material 3 — Chen et al. supplementary material [file S0033291725000893sup003.docx]

**Supplementary Methods**

**Recruitment and retention in ALSPAC**

Pregnant women resident in Avon, UK with expected dates of delivery between 1st April 1991 and 31st December 1992 were invited to take part in the study (Boyd et al., 2013; Fraser et al., 2013). The initial number of pregnancies enrolled was 14541, and 13988 children were alive at 1 year of age. The total sample size for analyses using any data collected after the age of seven is therefore 15447 pregnancies, of these 14901 children were alive at 1 year of age (Boyd et al., 2013; Fraser et a., 2013). 14203 unique mothers were initially enrolled in the study, and a further 630 women who did not enrol originally have provided data since their child was 7 years of age, this provides a total of 14,833 unique women (G0 mothers) enrolled in ALSPAC as of September 2021 (Major-Smith et al., 2023). 12113 G0 partners have been in contact with the study, and there are 3,807 G0 partners currently enrolled (Northstone et al., 2019). Children who were unable to join the study at age one were invited to participate in the study during adolescence and adulthood bringing the cumulative total to 15,546 participants.

***Exposure***

ALSPAC study measured 19 different ACEs, including 10 “classic” ACEs: physical abuse, sexual abuse, emotional abuse, emotional neglect, bullying, household violence, household substance use problems, parental mental health problems, parental convictions, and parental separation; and nine “extended” ACEs, including social class, financial difficulties, neighbourhood satisfaction, violence between child and partner, physical illness of child and parent, social support received by parent and child, and poor parent-child bond.

The definitions of the five ACEs used in this study are listed below:

- (1) physical abuse, defined as “*adult in family was ever physically cruel towards or hurt the child*”
- (2) sexual abuse, defined as the child was “*ever sexually abused, forced to perform sexual acts or touch someone in a sexual way*
- (3) emotional abuse, defined as “*parent was ever emotionally cruel towards the child or often said hurtful/insulting things to the child*”
- (4) emotional neglect, defined as “c*hild always felt excluded, misunderstood or never important to family, parents never asked or never listened when child talked about their free time*”
- (5) bullying, defined as “*child was a victim of bullying on a weekly basis*

***Outcome***

Three variables were needed for the pooled stage 1b+ mental health outcome:

- Depression was measured by the Clinical Interview Schedule-Revised (CIS-R).
  - Stage 1b+ depression is defined as having recurrent depression (i.e., moderate major depressive episode (MDE)) at both 18- and 24-year-old measurements, or severe MDE at either 18- or 24 years of age.
- Psychosis was examined by the Psychosis Like Symptoms (PLIKS), determined by self-report and computer-based interview.
  - Stage 1b+ psychosis was defined by having definite (at least) monthly psychotic symptoms including unrelated to sleep, fever, or drug use, and associated with distress, functional impairment or help-seeking in the past 6 months, at either 18- or 24-years of age.
- Anxiety was measured by the (CIS-R).
  - Stage 1b+ anxiety was defined by having recurrent anxiety at both 18- and 24-year-old measurement or having severe anxiety at either time point.

***Effect modifiers***

1. First-degree family history of severe mood and psychotic disorders, determined by parents’ self-report from birth until children were 8 years of age, or until parents were approximately 35 years of age:

- Family history of severe depression, defined as either parent having had severe depression, having seen doctors for depression, having received antidepressants, or having attempted suicide.
- Family history of schizophrenia, defined as either parent or parent’s biological parents having schizophrenia.

1. Sex, defined as biological sex measured at birth, as opposed to self-identified gender, which could be impacted by a wide range of sociobiological factors.
2. Personality scores are measured at age 13 years and 6 months, using the International Personality Item Pool, a 50-item scale that asks participants to choose the option that best describes how well each item describe them from “Very inaccurate”, “Moderately inaccurate”, “Neither inaccurate nor accurate”, “Moderately accurate”, and “Very accurate”. The items for each trait are:
   1. Intellect/openness markers include:
      1. Have a rich vocabulary.
      2. Have a vivid imagination.
      3. Have excellent ideas.
      4. Am quick to understand things.
      5. Use difficult words.
      6. Spend time reflecting on things.
      7. Am full of ideas.
      8. Have difficulty understanding abstract ideas.
      9. Am not interested in abstract ideas.
      10. Do not have a good imagination.
   2. Conscientiousness markers include:
      1. Am always prepared.
      2. Pay attention to details.
      3. Get chores done right away.
      4. Like order.
      5. Follow a schedule.
      6. Am exacting in my work.
      7. Leave my belongings around.
      8. Make a mess of things.
      9. Often forget to put things back in their proper place.
      10. Shirk my duties.
   3. Extraversion markers include:
      1. Am the life of the party.
      2. Feel comfortable around people.
      3. Start conversations.
      4. Talk to a lot of different people at parties.
      5. Don't mind being the centre of attention.
      6. Don't talk a lot.
      7. Keep in the background.
      8. Have little to say.
      9. Don't like to draw attention to myself.
      10. Am quiet around strangers.
   4. Agreeableness markers include:
      1. Am interested in people.
      2. Sympathize with others' feelings.
      3. Have a soft heart.
      4. Take time out for others.
      5. Feel others' emotions.
      6. Make people feel at ease.
      7. Am not really interested in others.
      8. Insult people.
      9. Am not interested in other people's problems.
      10. Feel little concern for others.
   5. Emotional stability markers include:
      1. Am relaxed most of the time.
      2. Seldom feel blue.
      3. Get stressed out easily.
      4. Worry about things.
      5. Am easily disturbed.
      6. Get upset easily.
      7. Change my mood a lot.
      8. Have frequent mood swings.
      9. Get irritated easily.
      10. Often feel blue.
3. Neurocognition, defined as full scale intelligence quotient (IQ) scores measured at age 8 years, measured by the Wechsler Intelligence Scale for Children Full-Scale IQ, which reflects the child’s general intellectual ability.

***Confounders***

1. Sex, defined as above.
2. Maternal age, defined as the age of the mother at the birth of the child, calculated from the mother’s date of birth and the date of delivery.
3. Child’s ethnicity, determined based on mother’s and mother’s partner’s and parents’ ethnicity through self-report.
   1. Mothers may describe her partner’s, parents’ and her ethnicity to be white, black/Caribbean, black/African, black/other (including Indian, Pakistani, Bangladeshi, Chinese, and other ethnic group), and the child’s ethnicity was determined as non-white if either their mother or mother’s partner’s ethnic background is not white, and white if both are white.
4. Social class, determined based on parental occupation.
   1. Mothers 32 weeks gestation were asked to report their partner’s and their occupation, where responses may include foreman, manager, supervisor, leading hand, self-employed, and none of these. Social class is determined by the highest of mother’s and partner’s occupation, dichotomised as high (e.g., professional/managerial) and low social class (e.g., unskilled) according to the Registrar General’s Social Classes.

**Auxiliary variables used for multiple imputation**

1. Violence between parents measured from when the child was between 0 to 16 years of age
2. Substance use in the household measured from when the child was between 0 to 16 years of age
3. Parental mental health problems measured from when the child was between 0 to 16 years of age
4. Parental conviction of offence measured from when the child was between 0 to 16 years of age
5. Parental separation measured from when the child was between 0 to 16 years of age
6. Financial difficulties measured from when the child was between 0 to 16 years of age
7. Neighbourhood satisfaction measured from when the child was between 0 to 16 years of age
8. Social support received by the child measured from when the child was between 0 to 16 years of age
9. Social support received by parents measured from when the child was between 0 to 16 years of age
10. Physical illness of the child measured from when the child was between 0 to 16 years of age
11. Physical illness of parents measured from when the child was between 0 to 16 years of age
12. Parent-child bond measured from when the child was between 0 to 16 years of age
13. Any behavioural disorder at 13 years of age, measured by the parent 6-band computer prediction, ICD-10 and DSM
14. Any behavioural disorder at 10 years of age, measured by the parent 6-band computer prediction, ICD-10 and DSM
15. Home ownership status
16. Mother’s highest education qualification
17. Edinburgh Postnatal Depression Scale (EDPS) score in habituation
18. Number of missing values in the Edinburgh Postnatal Depression Scale score in habituation
19. Frequency of alcohol use since birth
20. Mother’s opinion of neighbourhood in pregnancy
21. Crowding index in pregnancy
22. Major financial problem since pregnancy
23. Difficulty in affording accommodation in pregnancy
24. Major financial problem since mid-pregnancy
25. Difficulty affording food in pregnancy
26. Total rooms at home
27. EPDS Score in pregnancy
28. Number of missing values in EPDS Score in pregnancy

Study data were collected and managed using REDCap electronic data capture tools hosted at the University of Bristol (Harris et al., 2009). REDCap (Research Electronic Data Capture) is a secure, web-based software platform designed to support data capture for research studies.

**Interaction Continuum for Two Causative Exposures**

***Reproduced from VanderWeele 2019. The interaction continuum. Epidemiology 2019; 30: 648-656 (VanderWeele, 2019)***

- Rank 1: Positive multiplicative positive additive – synergistic interaction, indicating that the combined effect of the exposure and the effect modifier is greater than the product of their individual effects (multiplicative) and greater than the sum of their individual effects (additive)
- Rank 2: No multiplicative positive additive – purely additive interaction, indicating that the combined effect is the same as the product of their individual effects (no multiplicative interaction) but greater than the sum of their individual effects (positive additive
- Rank 3: Negative multiplicative positive additive – indicating that the combined effect is less than the product of their individual effects (negative multiplicative) but greater than the sum of their individual effects (positive additive)
- Rank 4: Negative multiplicative zero additive – indicating that that the combined effect is less than the product of their individual effects (negative multiplicative) and the same as the sum of their individual effects (zero additive)
- Rank 5: Negative multiplicative negative additive - indicating that the combined effect is less than both the product and the sum of their individual effects
- Rank 6: Single pure interaction for X_2_ - the interaction term contributes to the effect only for the second exposure, with no main effect
- Rank 7: Single qualitative interaction for X_2_ - the effect of X_2_ changes depending on the presence or level of the other exposure X_1_, suggesting a flip in the direction of the effect
- Rank 8: Pure interaction for X_1_, qualitative interaction for X_2_ - there is a pure interaction for X_1_, with no individual main effect, and a qualitative interaction for X_2_, with changing effect direction depending on conditions
- Rank 9: Double qualitative interaction - qualitative interactions for both X_1_ and X_2_, indicating complex and changing relationships depending on the presence or level of other variables
- Rank 10: Perfect antagonism - the combined effect is completely negated due to the interaction, indicating an extreme case of negative multiplicative and additive interaction
- Rank 11: Inverted interaction - the combined effect is opposite to what would be expected based on individual effects, suggesting a significant reversal due to the interaction term

**Supplemental References**

Boyd, A., Golding, J., Macleod, J., Lawlor, D. A., Fraser, A., Henderson, J., Molloy, L., Ness, A., Ring, S., & Davey Smith, G. (2013). Cohort Profile: the 'children of the 90s'--the index offspring of the Avon Longitudinal Study of Parents and Children. *International journal of epidemiology*, *42*(1), 111–127. <https://doi.org/10.1093/ije/dys064>

Fraser, A., Macdonald-Wallis, C., Tilling, K., Boyd, A., Golding, J., Davey Smith, G., Henderson, J., Macleod, J., Molloy, L., Ness, A., Ring, S., Nelson, S. M., & Lawlor, D. A. (2013). Cohort Profile: the Avon Longitudinal Study of Parents and Children: ALSPAC mothers cohort. *International journal of epidemiology*, *42*(1), 97–110. <https://doi.org/10.1093/ije/dys066>

Harris, P. A., Taylor, R., Thielke, R., Payne, J., Gonzalez, N., & Conde, J. G. (2009). Research electronic data capture (REDCap)--a metadata-driven methodology and workflow process for providing translational research informatics support. Journal of biomedical informatics, 42(2), 377–381. <https://doi.org/10.1016/j.jbi.2008.08.010>

Major-Smith, D., Heron, J., Fraser, A., Lawlor, D. A., Golding, J., & Northstone, K. (2023). The Avon Longitudinal Study of Parents and Children (ALSPAC): a 2022 update on the enrolled sample of mothers and the associated baseline data. *Wellcome open research*, *7*, 283. <https://doi.org/10.12688/wellcomeopenres.18564.1>

Northstone, K., Lewcock, M., Groom, A., Boyd, A., Macleod, J., Timpson, N., & Wells, N. (2019). The Avon Longitudinal Study of Parents and Children (ALSPAC): an update on the enrolled sample of index children in 2019. *Wellcome open research*, *4*, 51. <https://doi.org/10.12688/wellcomeopenres.15132.1>

VanderWeele T. J. (2019). The Interaction Continuum. *Epidemiology (Cambridge, Mass.)*, *30*(5), 648–658. https://doi.org/10.1097/EDE.0000000000001054
